# Measurement of SARS-CoV-2 Antibody Titers Improves the Prediction Accuracy of COVID-19 Maximum Severity by Machine Learning in Non-Vaccinated Patients

**DOI:** 10.3389/fimmu.2022.811952

**Published:** 2022-01-21

**Authors:** Makoto Kurano, Hiroko Ohmiya, Yoshiro Kishi, Jun Okada, Yuki Nakano, Rin Yokoyama, Chungen Qian, Fuzhen Xia, Fan He, Liang Zheng, Yi Yu, Daisuke Jubishi, Koh Okamoto, Kyoji Moriya, Tatsuhiko Kodama, Yutaka Yatomi

**Affiliations:** ^1^ Department of Clinical Laboratory, The University of Tokyo Hospital, Tokyo, Japan; ^2^ Department of Clinical Laboratory Medicine, Graduate School of Medicine, The University of Tokyo, Tokyo, Japan; ^3^ Business Planning Department, Sales & Marketing Division, Medical & Biological Laboratories Co., Ltd, Tokyo, Japan; ^4^ The Key Laboratory for Biomedical Photonics of Ministry of Education at Wuhan National Laboratory for Optoelectronics - Hubei Bioinformatics & Molecular Imaging Key Laboratory, Systems Biology Theme, Department of Biomedical Engineering, College of Life Science and Technology, Huazhong University of Science and Technology, Wuhan, China; ^5^ Reagent R&D Center, Shenzhen YHLO Biotech Co., Ltd, Shenzhen, China; ^6^ Department of Infection Control and Prevention, The University of Tokyo, Tokyo, Japan; ^7^ Laboratory for Systems Biology and Medicine, Research Center for Advanced Science and Technology, The University of Tokyo, Tokyo, Japan

**Keywords:** COVID-19, severity, machine learning, IgM, IgG, IgA, nucleocapsid protein, spike protein

## Abstract

Numerous studies have suggested that the titers of antibodies against SARS-CoV-2 are associated with the COVID-19 severity, however, the types of antibodies associated with the disease maximum severity and the timing at which the associations are best observed, especially within one week after symptom onset, remain controversial. We attempted to elucidate the antibody responses against SARS-CoV-2 that are associated with the maximum severity of COVID-19 in the early phase of the disease, and to investigate whether antibody testing might contribute to prediction of the disease maximum severity in COVID-19 patients. We classified the patients into four groups according to the disease maximum severity (severity group 1 (did not require oxygen supplementation), severity group 2a (required oxygen supplementation at low flow rates), severity group 2b (required oxygen supplementation at relatively high flow rates), and severity group 3 (required mechanical ventilatory support)), and serially measured the titers of IgM, IgG, and IgA against the nucleocapsid protein, spike protein, and receptor-binding domain of SARS-CoV-2 until day 12 after symptom onset. The titers of all the measured antibody responses were higher in severity group 2b and 3, especially severity group 2b, as early as at one week after symptom onset. Addition of data obtained from antibody testing improved the ability of analysis models constructed using a machine learning technique to distinguish severity group 2b and 3 from severity group 1 and 2a. These models constructed with non-vaccinated COVID-19 patients could not be applied to the cases of breakthrough infections. These results suggest that antibody testing might help physicians identify non-vaccinated COVID-19 patients who are likely to require admission to an intensive care unit.

## Introduction

Coronavirus disease 2019 (COVID-19), caused by severe acute respiratory syndrome coronavirus 2 (SARS-CoV-2), exhibits a wide clinical spectrum, ranging from an asymptomatic state to severe disease requiring mechanical respiratory support. For proper triage of the patients and appropriate use of medical resources for patients with COVID-19, it is important to identify suitable biomarkers/diagnostic systems for predicting the maximum severity of COVID-19 in the early phase of the disease. Until date, several demographic characteristics and clinical features, including laboratory data, have been reported to be associated with the severity of COVID-19, including male sex, advanced age, underlying hypertension, diabetes, and cardiovascular disease, positive smoking history (1, 2), and serum CRP and D-Dimer levels (3, 4).

In addition to the aforementioned parameters, the titers of antibodies against SARS-CoV-2 have also been reported by several studies to be associated with the disease severity in COVID-19 patients. While, in general, studies have reported positive associations between SARS-CoV-2 antibody titers and the clinical disease severity and/or laboratory data such as the serum CRP, a few studies have denied the existence of a positive association between the antibody titers and the severity of COVID-19 (5–7). The timing of measurement of the antibodies varied among these studies; while the measurements were made in a rather acute phase of the disease, that is, within 2 weeks from the onset of symptoms, in some studies (8–20), in others, they were made in the later phases of the disease (8–10, 13, 16–19, 21–32), at the time of admission to the hospital (33, 34), or at arbitrary times (35–37). Previous studies have demonstrated positive associations between the clinical severity of COVID-19 and variously measured antibody responses, including the neutralizing antibody titers (8, 10, 17, 20, 21, 26, 28, 31, 34–37), total antibody titers (9, 27, 36), IgG titers (10, 12–14, 16, 18, 22, 28–30, 32, 33), IgM titers (10, 11, 22–24, 32), and/or IgA titers (10, 13, 18, 19, 22, 29, 32). Various antigens eliciting the antibody responses have been also demonstrated to be associated with the disease severity, including antibodies elicited against the spike (S) protein and/or receptor-binding domain (RBD) in the S protein (12, 16, 19, 22, 29, 30, 32, 36), antibodies elicited against the nucleocapsid (N) protein (10, 11, 13, 23, 32, 33, 36), and antibodies against both the S and N proteins (14, 24).

While the aforementioned observational studies demonstrated positive associations of the antibody titers with the disease severity, several issues still remain to be resolved. For example, since many of these studies did not measure the IgG, IgM, and IgA titers against the S protein, RBD, or N protein simultaneously, it remains unclear as to which of these are associated with the maximum severity of COVID-19. Moreover, while antibody testing undoubtedly contributes to the diagnosis of COVID-19 (38), it is necessary to clarify whether antibody testing could also contribute to prediction of the maximum severity of COVID-19, in order to establish its usefulness in clinical practice. In most previous studies, the antibody titers were not measured serially at short intervals, for example once in a week, even though they could be expected to change dynamically, especially in the early phase of the disease.

**Figure 1:**
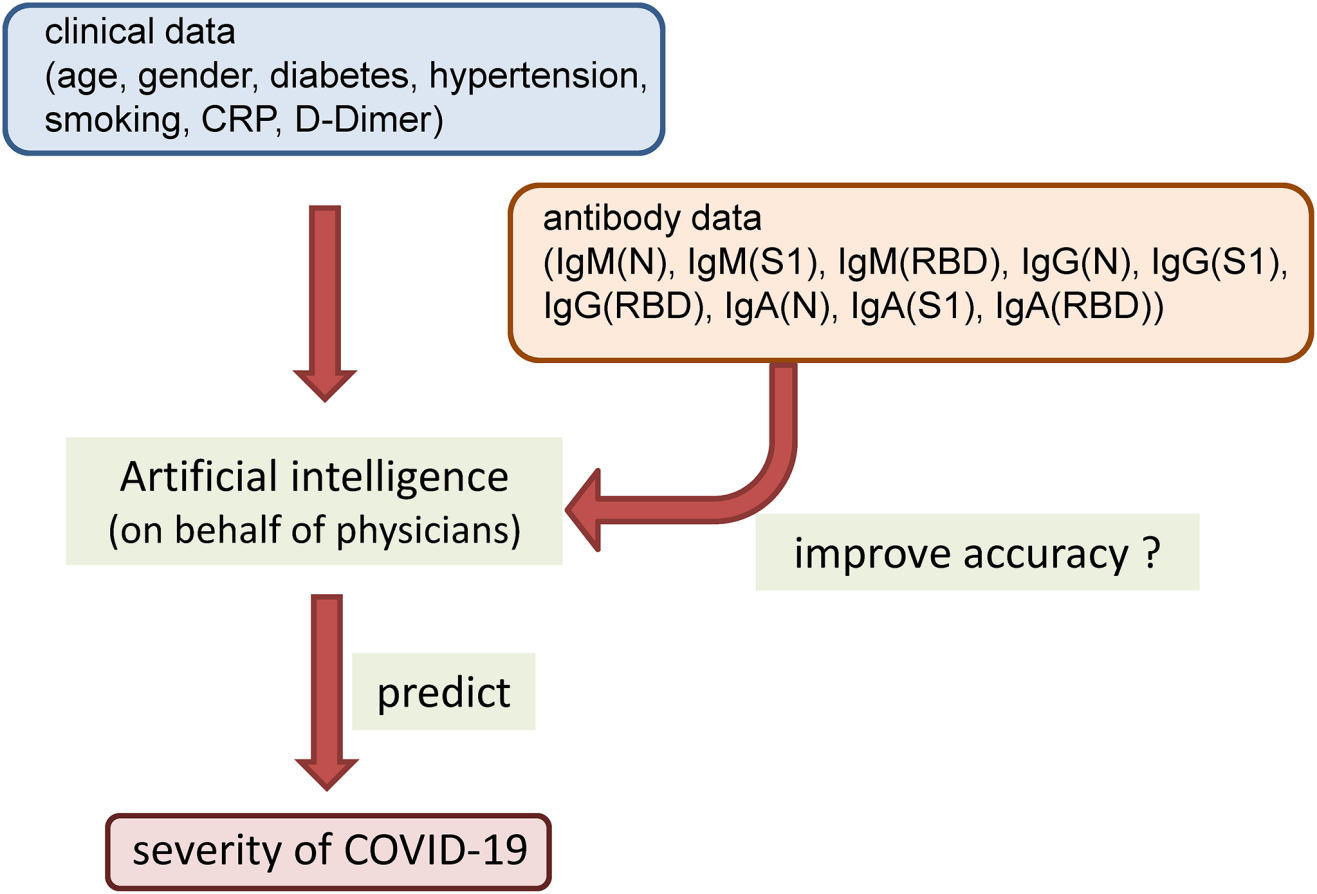
The concept for using a machine learning approach in the present study.

To resolve these issues related to determining the usefulness of antibody testing for prediction of the maximum severity of COVID-19, we attempted to find answers to the following questions by the study approaches described below. (1) What types of antibody responses to SARS-CoV-2 that are associated with the maximum severity of COVID-19 are elicited in the early phase of the disease? To answer this question, we serially measured the titers of IgM, IgG, and IgA elicited against the N protein, S1 protein, and RBD simultaneously in samples collected within short intervals of one or two days until 12 days after symptom onset and compared the titers among four patient groups classified according to the disease maximum severity: severity group 1 (did not require oxygen supplementation), severity group 2a (required oxygen supplementation at low flow rates of under 4 L/min *via* a nasal cannula), severity group 2b (required oxygen supplementation at relatively high flow rates, but not mechanical ventilatory support), and severity group 3 (required mechanical ventilatory support). (2) Does antibody measurement contribute to prediction of the disease maximum severity in patients with COVID-19? To answer this question, we used an artificial intelligence, on behalf of thinking of physicians, to answer this question objectively. We constructed models using a machine learning approach based on clinical and laboratory parameters with or without addition of the results of antibody testing and investigated whether addition of the antibody data improved the ability of the machine learning models to predict the disease maximum severity in COVID-19 patients (**Figure 1**).

## Methods

### Samples

We collected the residual serum samples after routine clinical testing of 134 subjects who had been diagnosed as having COVID-19 by RT-PCR assay between April 2020 and January 2021. None of the subjects had been vaccinated against SARS-CoV-2. The subjects were classified into four groups according to the disease maximum severity: severity group 1 (did not require oxygen supplementation), severity group 2a (required oxygen supplementation at low flow rates of under 4 L/min *via* a nasal cannula), severity group 2b (required oxygen supplementation at relatively high flow rates, but not mechanical ventilatory support), and severity group 3 (required mechanical ventilatory support). We subclassified patients of severity group 2 into groups 2a and 2b, since the clinical phenotypes and necessity of admission to the intensive care unit were quite different between these two subgroups. The characteristics of the subjects are described in **Supplemental Table S1**. To investigate whether the models could be applied to the cases of breakthrough infections, we used 33 points of clinical and antibody data obtained from 11 individual subjects. Two of the subjects had taken mRNA vaccine twice and others had taken once. The average duration from the last vaccination to the onset of symptom was 10 days for the patients who had taken vaccination once and 16 days for those who had taken twice.

The current study was performed in accordance with the ethical guidelines laid down in the Declaration of Helsinki. Written informed consent for sample analysis was obtained from some of the patients. For the remaining participants from whom written informed consent could not be obtained (owing to their having been discharged or transferred out of the hospital), informed consent was obtained in the form of an opt-out on the website, as follows. Patients were informed about the study on the website and those who were unwilling to be enrolled in our study were excluded. The study design was approved by The University of Tokyo Medical Research Center Ethics Committee (2019300NI-4 and 2020206NI).

### Measurements of Antibodies Against SARS-CoV-2

Antibody testing was performed using an iFlash3000 fully automated chemiluminescent immunoassay analyzer (Shenzhen YHLO Biotech Co., Ltd., China). The assay procedure adopted was in accordance with that described by Qian C, et al. (39), with minor modifications. Briefly, acridinium-labeled anti-human IgM, IgG, or IgA conjugate antibody was used to detect the antibodies bound to the beads. The magnetic beads used in these chemiluminescent immunoassays were coated with each of the antigens of SARS-CoV-2 (N protein, S1 protein, or RBD). The SARS-CoV-2 IgM, IgG, or IgG titers in 5-uL samples were calculated in relative light units (RLU) obtained from the analyzer and expressed as arbitrary units per milliliter (AU/mL), by comparing the RLU detected by the iFlash optical system with the cutoff calculated from the calibrators containing anti-SARS-CoV-2 IgM, IgG, or IgA chimeric antibody.

### Statistical Analysis

**Figure 2:**
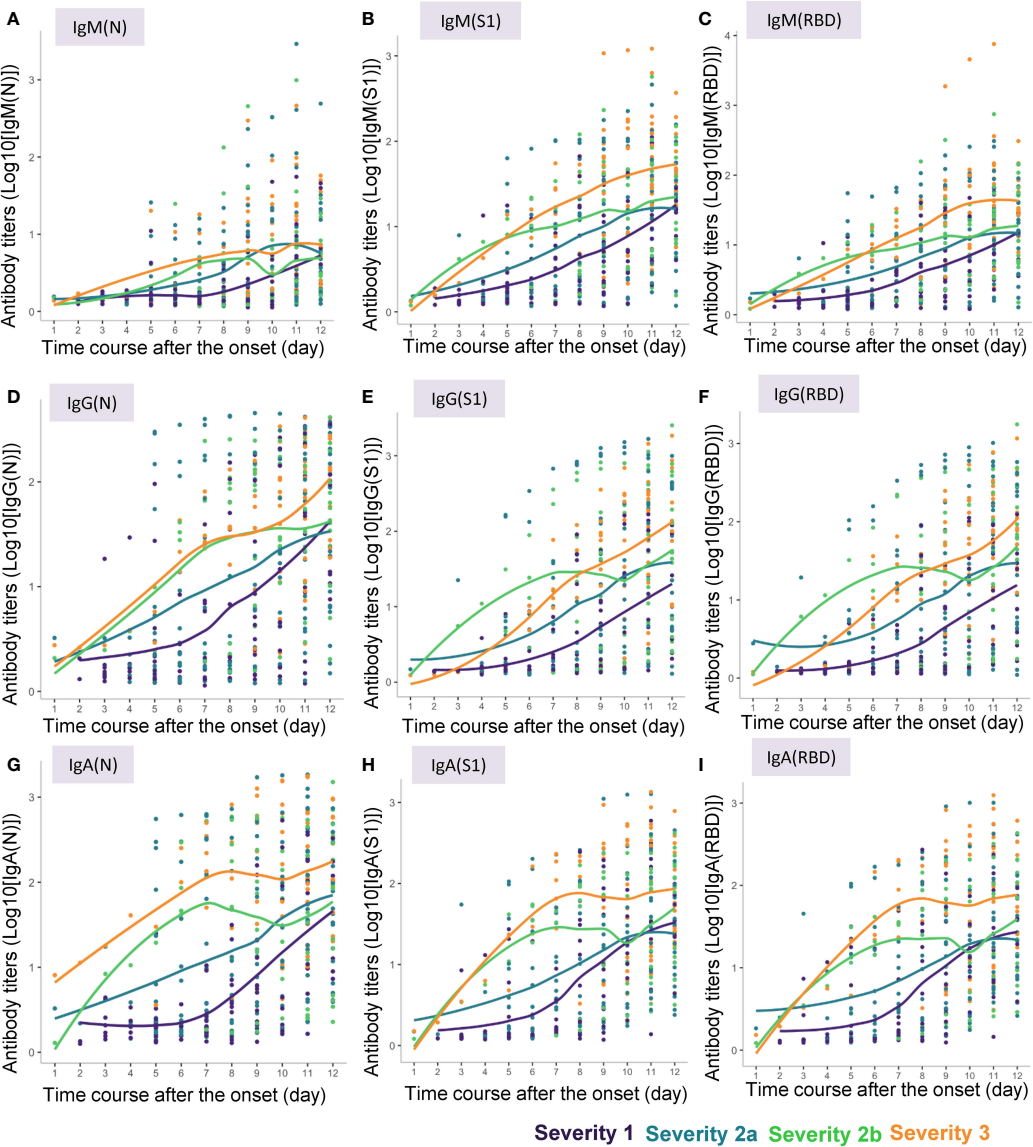
Approximate curves for the antibody kinetics in COVID-19 patients classified by the disease maximum severity. Local polynomial regression curves were fitted to indicate the antibody responses to the COVID-19 antigens until day 12 after symptom onset in the patients with different maximum severity levels of COVID-19. **(A)** IgM(N), **(B)** IgM(S1), **(C)** IgM(RBD), **(D)** IgG(N), **(E)** IgG(S1), **(F)** IgG(RBD), **(G)** IgA(N), **(H)** IgA(S1), **(I)** IgA(RBD).

Locally weighted scatter plot smoothing (LOESS) lines were fitted to visualize the changes in the antibody responses to the COVID-19 antigens over time in COVID-19 patients of the four severity groups (**Figure 2**). These lines were plotted by the ggplot2 package (version 3.3.5) in the R language.

The antibody responses to the COVID-19 antigens measured at different time-points after symptom onset were compared among the four severity groups. The Brunner-Munzel test (40) was used to analyze the differences after verifying the significant deviations from normality and homoscedasticity of the datasets by the F and Shapiro-Wilk tests (41).

Machine learning models were developed to predict the maximum severity of the disease in the subjects based on the clinical information, including the age, gender, presence/absence of underlying diabetes mellitus and hypertension, current smoking history, serum levels of CRP and D-Dimer, and the results of antibody testing. Out of the 111 cases used to develop the machine learning model, multiple blood samples had been obtained between 4 to 12 days after symptom onset in most subjects. In total, 316 samples with complete measurements of features were collected. To explore whether the antibody data obtained in the early phase of the disease could improve the prediction accuracy of the models, 6 subsets were created according to the timings of the sample collection: from 4 to 7 days (day 4-7), 5 to 8 days (day 5-8), 6 to 9 days (day 6-9), 7 to 10 days (day 7-10), 8 to 11 days (day 8-11), and 9 to 12 days (day 9-12) after symptom onset. These subsets were randomly split into training (70%) and validation (30%) datasets with stratification sampling for the severity group as the label using the initial split function of the R language. Since the frequency of the severity groups was unbalanced, that is, the number of samples in one group was much higher than that in another group (**Supplemental Table S2**), class weights calculated by the following formula were set to groups to penalize the misclassification:

$w_{j}=\frac{N}{ng\;\times \;n_{j}}$ where *w_j_* is the weight in group j (j = 1 ~ *ng*; *ng* is the number of groups), *N* is the total number of samples, and *n_j_* is number of samples from group j. In the training phase, XGBoost (eXtreme Gradient Boosting) classifier (42) models were optimized by tuning hyperparameters and repeating 3-fold cross-validation. The optimum hyperparameters for each model were found by grid search, and are described in **Supplemental Table S3**.　The XGBoost classifies samples into several categories based on a trained gradient boosting decision tree and has been used for similar studies (43–45). To investigate the impact of antibody titers as features on the model accuracy, models with and without inclusion of the results of antibody testing as input data were built, and the feature importance was calculated.

*P <*0.05 was regarded as denoting statistical significance in all the analyses.

## Results

### The Antibody Response to SARS-CoV-2 Was Differently Influenced by the Maximum Severity of COVID-19

We measured the serum levels of IgM, IgG, and IgA against the N protein (IgM(N), IgG(N), and IgA(N)), S1 protein (IgM(S1), IgG(S1), and IgA(S1)), and RBD (IgM(RBD), IgG(RBD), and IgA(RBD)) in the serum samples of the COVID-19 patients collected until 12 days after symptom onset. The approximate curves, drawn from the results in the subjects classified according to the maximum severity of COVID-19, are shown in **Figure 2**, and those of the ratios of IgM(S1) to IgM(N) (IgM(S1/N)), IgM(RBD) to IgM(N) (IgM(RBD/N)), IgM(RBD) to IgM(S1) (IgM(RBD/S1)), IgG(S1) to IgG(N) (IgG(S1/N)), IgG(RBD) to IgG(N) (IgG(RBD/N)), IgG(RBD) to IgG(S1) (IgG(RBD/S1)), IgA(S1) to IgA(N) (IgA(S1/N)), IgA(RBD) to IgA(N) (IgA(RBD/N)), and IgA(RBD) to IgA(S1) (IgA(RBD/S1)) are shown in **Supplemental Figure S1**.

In general, the absolute titers of the antibodies increased with increasing severity level of COVID-19. In regard to the kinetics of IgM, “the titers of all of IgM(N), IgM(S1) and IgM(RBD) seemed to increase as the disease maximum severity increased. Especially, the titers of IgM(N) increased earlier in severity group 2a or greater than in severity group 1, while the titers of IgM(S1) and IgM(RBD) increased earlier in severity groups 2b and 3 than in severity groups 1 and 2a (**Figures 2A–C**). No obvious differences were observed in the time-course of changes in the IgM(S1/N), IgM(RBD/N), and IgM(RBD/S1) among the four severity groups, except that the IgM(S1/N) and IgM(RBD/N) seemed to be higher around day 4 in severity group 2b than in the other severity groups (**Supplemental Figures S1A–C**). In regard to the kinetics of IgG, the titers of IgG(S1) and IgG(RBD) increased in a bell-shaped manner depending on the disease maximum severity from day 3 to day 6; the IgG(S1) and IgG(RBD) titers appeared to increase earlier in severity group 2b than in severity groups 1, 2a, and 3, while the time-course of increase of the IgG(N) titers appeared to be similar between severity groups 2b and 3 (**Figures 2D–F**). IgG(S1/N) and IgG(RBD/N) appeared to be higher in severity group 2b, and lower in severity group 3, as compared to the ratios in severity groups 1 and 2a (**Supplemental Figures S1D–F**). In regard to the kinetics of IgA, the titers of IgA increased with increasing maximum severity of COVID-19, especially from day 3 to day 6, and no differences were observed in the pattern of increase of IgA(N), IgA(S1) and IgA(RBD) among the severity group (**Figures 2G–I**). No obvious differences in the IgA(S1/N), IgA(RBD/N) or IgA(RBD/S1) were observed among the four severity groups, except that IgA(S1/N) and IgA(RBD/N) seemed rather higher in severity group 1 (**Supplemental Figures S1G–I**).

### Cross-Sectional Analyses Revealed That the Antibody Titers Increased Significantly More Rapidly in Patients With More Severe Disease From as Early as Day 4-5 or Day 6-7 After Symptom Onset

**Figure 3:**
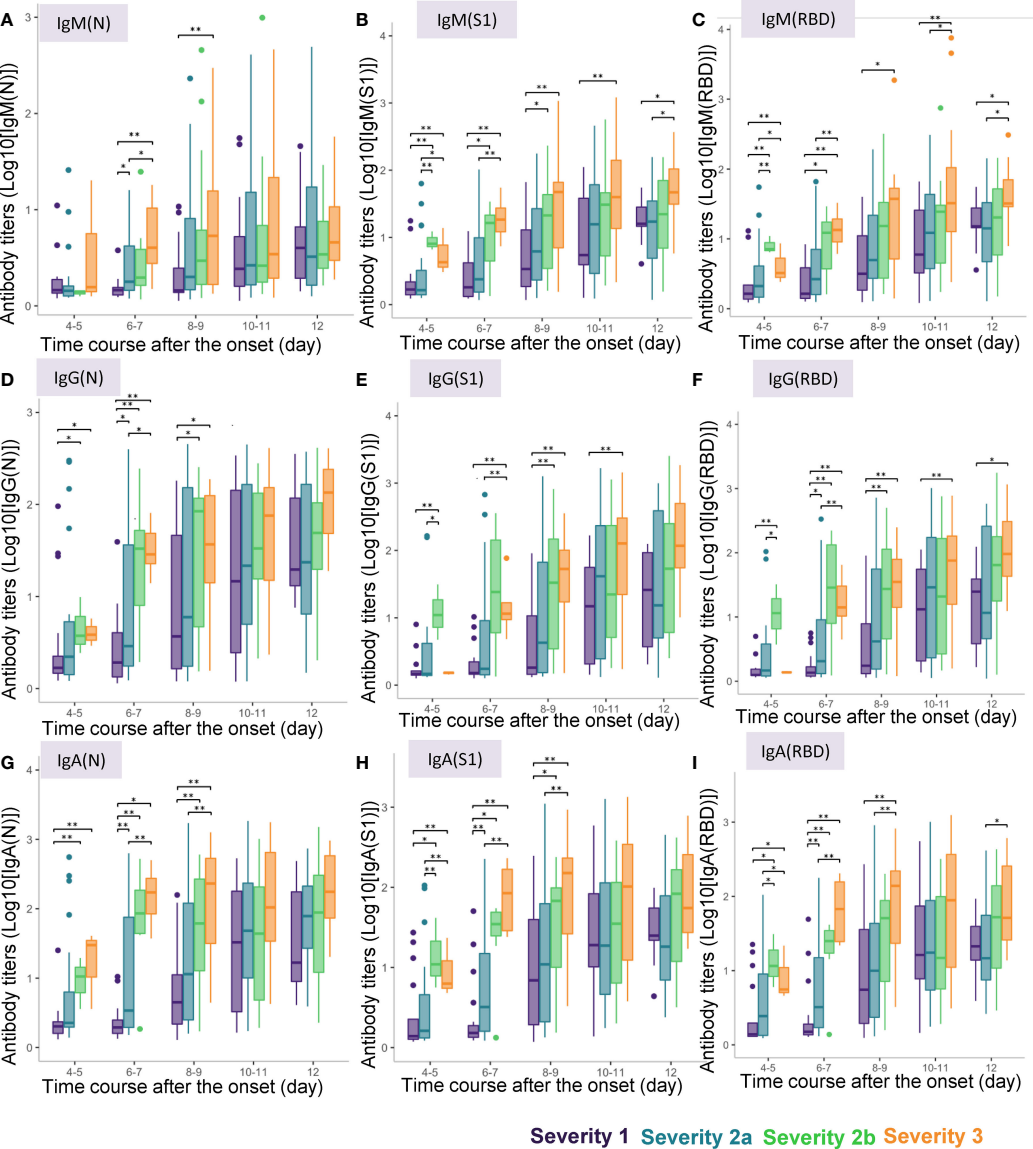
Differences in antibody titers among COVID-19 patients classified by the disease maximum severity. We compared the titers of different types of antibodies among COVID-19 patients classified according to the disease maximum severity as described in the *Material and Methods* section, measured on day 4-5, day 6-7, day 8-9, day 10-11, day 12 after symptom onset. *P < 0.05, **P < 0.01. The horizontal bar represents the median, the box bar represents the lower and upper quartiles, and the fine bar represents the minimum and maximum. **(A)** IgM(N), **(B)** IgM(S1), **(C)** IgM(RBD), **(D)** IgG(N), **(E)** IgG(S1), **(F)** IgG(RBD), **(G)** IgA(N), **(H)** IgA(S1), **(I)** IgA(RBD).

When we conducted a cross-sectional analysis of the antibody titers, significant differences were observed from day 4-5 after symptom onset in the titers of all the antibody responses, except for that of IgM(N), which began to show a significant increase only from day 6-7 (**Figure 3**). Although the titers of IgM(N), IgG(N), IgG(RBD), IgA(N), IgA(S1), and IgA(RBD) differed significantly between severity groups 1 and 2a, even larger differences were observed between severity groups 2a and 2b, and at more time-points. The duration after symptom onset until when significant differences were observed differed among the antibody types. While significant differences in the titers of IgM (N), IgG(N), IgA(N) and IgA(S1) among the four severity groups were observed until day 8-9, differences in the other antibody responses were observed until day 12. These results suggest the potential usefulness of antibody testing to identify severity group 2b and 3 patients even at a rather early phase of the disease (that is, by day 8-9).

Interestingly, in regard to the ratios of the antibody titers, while significant differences were found in the IgM (IgM(S1/N), IgM(RBD/N) and IgM(RBD/S1) and IgG (IgG(S1/N), IgG(RBD/N) and IgG(RBD/S1)) ratios on day 3-4 and day 5-6 among the four severity groups, no such differences were found in the IgA ratios (**Supplemental Figure S2**). In regard to the ratios of the antibody titers against S1 protein/RBD to those against the N protein, IgM(S1/N), IgM(RBD/N), IgG(S1/N), and IgG(RBD/N) were higher only in severity group 2b, but not in severity group 3 on day 4-5. As for the ratios of the antibody titers against RBD to those against S1 protein, IgM(RBD/S1) was lower in severity group 3 on day 4-5 and day 6-7, and IgG(RBD/S1) was higher in severity group 3 on day 6-7. These results suggest the potential usefulness of measuring the antibody ratios to identify severity group 2b patients on day 4-5.

### Antibody Tests Did Not Improve the Ability of the Models Constructed Using a Machine Learning Technique to Distinguish Severity Groups 2a and Over From Severity Group 1

Lastly, we investigated whether the results of antibody testing could contribute to prediction of the disease maximum severity by a machine learning approach. We randomly divided the subjects into a training set and a validation set as described in the *Methods* section. We investigated two possible models: severity group 1 vs. severity groups 2a, 2b, and 3 (model 1), and severity groups 1 and 2a vs. severity groups 2b and 3 (model 2) and created the workflow with only clinical data or with both clinical data and antibody data. The analyses were performed with data obtained on day 4-7, day 5-8, day 6-9, day 7-10, day 8-11, and day 9-12 after symptom onset, considering that the disease onset was determined from a rather subjective disease history obtained from the subjects.

**Figure 4:**
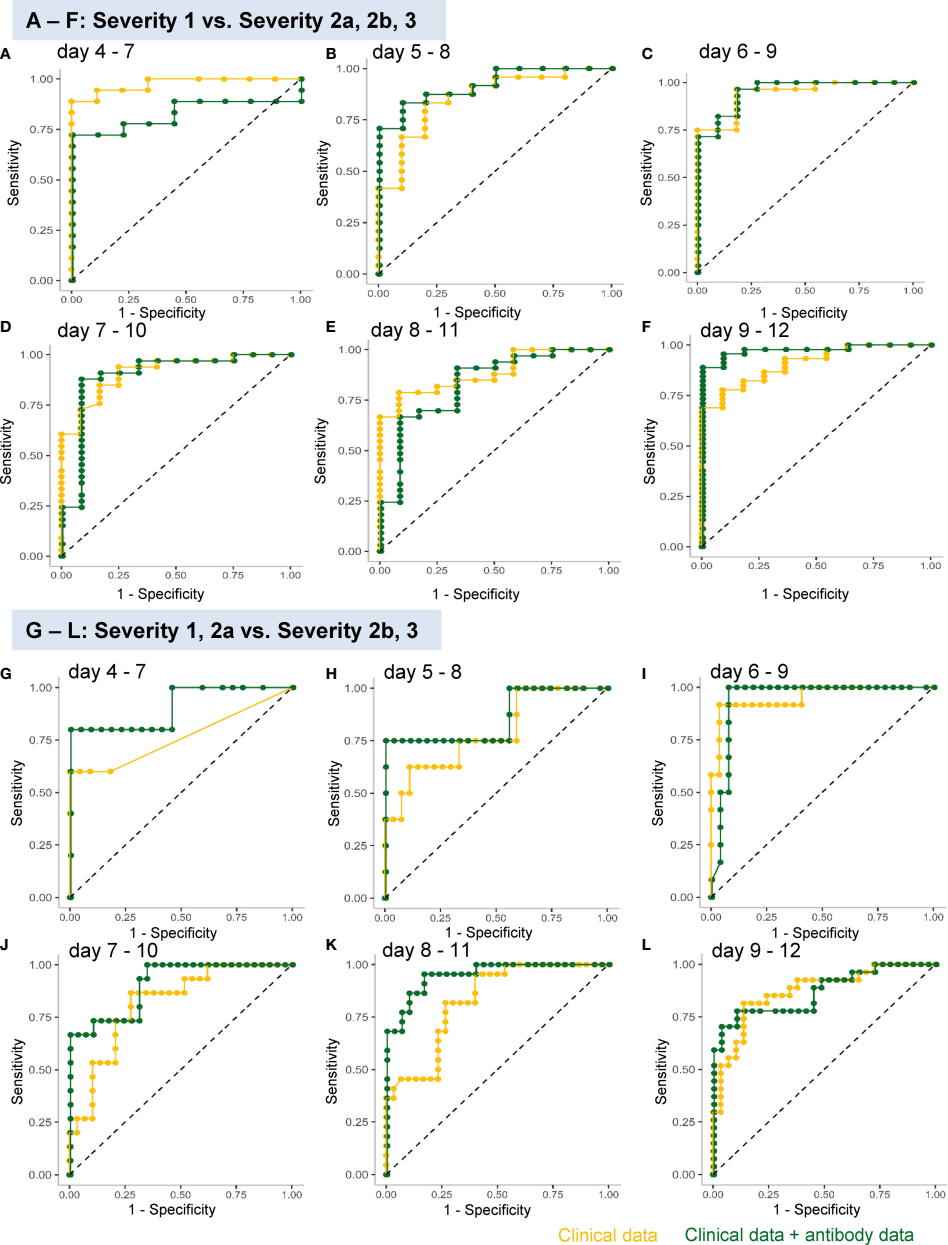
ROC analyses of the analysis models constructed using a machine learning technique for predicting the maximum severity of COVID-19. The ROCs of the analysis models constructed using a machine learning technique for predicting the COVID-19 severity, using the data obtained on day 4–7 **(A, G)**, day 5–8 **(B, H)**, day 6–9 **(C, I)**, day 7–10 **(D, J)**, day 8–11 **(E, K)**, and day 9–12 **(F, L)**, are shown. The models were constructed to distinguish severity groups 2a or over from severity group 1 **(A–F)** or distinguish severity groups 2b and 3 from severity groups 1 and 2a **(G–L)**. The yellow curves represent the ROCs of the model constructed using clinical parameters and the green curves represent those of the model constructed using both clinical and antibody data.

In regard to model 1, the workflow to predict the maximum severity which represents one of tree estimators in the optimum model on day 4-7 is shown in **Supplemental Figure S3** and the feature importance in the models is described in **Supplemental Figure S4**. The accuracy of the model in the validation set is shown in **Table 1**. As shown in the table, the addition of antibody data to the clinical parameters did not improve the ability of the model constructed by the machine learning technique to distinguish severity group 2a or over from severity group 1; in fact, it was worse based on the day 4-7 data. The receiver-operating characteristic (ROC) of the constructed models are shown in **Figures 4A–F**, which also did not suggest the usefulness of addition of antibody data to distinguish severity group 2a or over from severity group 1.

**Table 1:** The accuracy of the model to distinguish severity group 2a or over from severity group 1 in the validation set.

A. Clinical data alone					
Day	True Severity	Estimated S1 (n)	Estimate S2a, 2b, 3 (n)	Error Rate	Accuracy
day 4-7	S1 (n)	8	1	0.11	0.93
	S2a, 2b, 3 (n)	1	17	0.06	
day 5-8	S1 (n)	5	5	0.50	0.82
	S2a, 2b, 3 (n)	1	23	0.04	
day 6-9	S1 (n)	9	2	0.18	0.92
	S2a, 2b, 3 (n)	1	27	0.04	
day 7-10	S1 (n)	9	3	0.25	0.89
	S2a, 2b, 3 (n)	2	31	0.06	
day 8-11	S1 (n)	6	6	0.50	0.76
	S2a, 2b, 3 (n)	5	28	0.15	
day 9-12	S1 (n)	7	4	0.36	0.88
	S2a, 2b, 3 (n)	3	42	0.07	
**B. Clinical data + antibody data**					
**Day**	**True Severity**	**Estimated S1 (n)**	**Estimate S2a, 2b, 3 (n)**	**Error rate (%)**	**Accuracy**
day 4-7	S1 (n)	6	3	0.33	0.74
	S2a, 2b, 3 (n)	4	14	0.22	
day 5-8	S1 (n)	5	5	0.50	0.85
	S2a, 2b, 3 (n)	0	24	0.00	
day 6-9	S1 (n)	8	3	0.27	0.92
	S2a, 2b, 3 (n)	0	28	0.00	
day 7-10	S1 (n)	8	4	0.33	0.89
	S2a, 2b, 3 (n)	1	32	0.03	
day 8-11	S1 (n)	5	7	0.58	0.82
	S2a, 2b, 3 (n)	1	32	0.03	
day 9-12	S1 (n)	7	4	0.36	0.91
	S2a, 2b, 3 (n)	1	44	0.02	

### Antibody Tests Improved the Ability of the Models Constructed Using a Machine Learning Technique to Distinguish Severity Groups 2b and 3 From Severity Groups 1 and 2a

In regard to model 2, which was aimed at distinguishing severity groups 2b and 3 from severity groups 1 and 2a, the workflow to predict the maximum severity which represents one of tree estimators in the optimum model on day 4-7 is shown in **Supplemental Figure S5** and the feature importance in the models is described in **Supplemental Figure S6**. The accuracy of the model in the validation set is shown in **Table 2**. As shown in the table, the addition of antibody data to the clinical parameters improved the ability of the model constructed using a machine learning technique to distinguish severity groups 2b and 3 from severity groups 1 and 2a, especially based on the data of day 5-8, day7-10, and day8-11. It is important for physicians to avoid underestimating the disease maximum severity in severity groups 2b and 3, as these patients require treatment in an intensive care unit. In regard to the error rate in predicting the disease maximum severity in severity groups 2b and 3, the error rate was suppressed to a great degree, especially when the determination was made based on data obtained on day 4-7, day 5-8, and day 6-9. The ROC analyses also revealed that the addition of antibody data improved the predictive ability of the models, except for the model using data obtained on day 8-12 (**Figures 4G–L**).

**Table 2:** The accuracy of the model to distinguish severity groups 2b and 3 from severity groups 1 and 2a in the validation set.

A. Clinical data alone					
Day	True Severity	Estimated S1, 2a (n)	Estimate S2b, 3 (n)	Error Rate (%)	Accuracy
day 4-7	S1, 2a (n)	22	0	0.00	0.93
	S2b, 3 (n)	2	3	0.40	
day 5-8	S1, 2a (n)	25	2	0.07	0.80
	S2b, 3 (n)	5	3	0.63	
day 6-9	S1, 2a (n)	26	1	0.04	0.92
	S2b, 3 (n)	2	10	0.17	
day 7-10	S1, 2a (n)	26	3	0.10	0.77
	S2b, 3 (n)	7	8	0.47	
day 8-11	S1, 2a (n)	22	8	0.27	0.77
	S2b, 3 (n)	4	18	0.18	
day 9-12	S1, 2a (n)	25	4	0.14	0.84
	S2b, 3 (n)	5	22	0.23	
**B. Clinical data + antibody data**					
**Day**	**True Severity**	**Estimated S1 (n)**	**Estimate S2a, 2b, 3 (n)**	**Error Rate (%)**	**Accuracy**
day 4-7	S1, 2a (n)	22	0	0.00	0.96
	S2b, 3 (n)	1	4	0.20	
day 5-8	S1, 2a (n)	26	1	0.04	0.91
	S2b, 3 (n)	2	6	0.25	
day 6-9	S1, 2a (n)	25	2	0.07	0.95
	S2b, 3 (n)	0	12	0.00	
day 7-10	S1, 2a (n)	29	0	0.00	0.89
	S2b, 3 (n)	5	10	0.33	
day 8-11	S1, 2a (n)	25	5	0.17	0.88
	S2b, 3 (n)	1	21	0.05	
day 9-12	S1, 2a (n)	26	3	0.10	0.84
	S2b, 3 (n)	6	21	0.22	

When we analyzed the data with sub-grouping the subjects on day 1-6 and day 7-12, no obvious improvement of the predicting accuracy was observed in both models (**Supplemental Figures S7** – **S9** and **Supplemental Tables S4**, **S5**). These results suggest that the monitoring antibody titers in a narrow span is necessary to predict the maximum severity of COVID-19, since the antibody titers dramatically fluctuate as shown in **Figures 2** and **3**.

### The Models for the Prediction of Maximum Severity Constructed With the Data Obtained From Non-Vaccinated Patients Could Not Be Applied to the Cases of Breakthrough Infections

Since breakthrough infections are now clinical concerns, we lastly investigated whether the models for the prediction of COVID-19 maximum severity, which we had constructed with the data obtained from non-vaccinated patients, might help physicians to predict the maximum severity in the cases of breakthrough infections. As shown in **Tables 3** and **4**, antibody tests did not apparently improve the ability of the models constructed using a machine learning technique to distinguish maximum severity in both models, except the ability to distinguish severity group 2a or over from severity group 1 on day 4-7 in the cases of breakthrough infections, in comparison to the model constructed with clinical data alone.

**Table 3:** The accuracy of the model to distinguish severity group 2a or over from severity group 1 in the cases of breakthrough infection.

A. Clinical data alone					
Day	True Severity	Estimated S1 (n)	estimate S2a, 2b, 3 (n)	Error Rate	Accuracy
day 4-7	S1 (n)	3	2	0.40	0.78
	S2a, 2b, 3 (n)	0	4	0.00	
day 5-8	S1 (n)	3	1	0.25	0.90
	S2a, 2b, 3 (n)	0	6	0.00	
day 6-9	S1 (n)	1	2	0.33	0.78
	S2a, 2b, 3 (n)	0	6	0.00	
day 7-10	S1 (n)	1	2	0.67	0.75
	S2a, 2b, 3 (n)	0	5	0.00	
day 8-11	S1 (n)	1	3	0.75	0.63
	S2a, 2b, 3 (n)	1	6	0.14	
day 9-12	S1 (n)	1	3	0.75	0.60
	S2a, 2b, 3 (n)	1	5	0.17	
**B. Clinical data + antibody data**					
**Day**	**True Severity**	**Estimated S1 (n)**	**Estimate S2a, 2b, 3 (n)**	**Error Rate (%)**	**Accuracy**
day 4-7	S1 (n)	4	1	0.20	0.89
	S2a, 2b, 3 (n)	0	4	0.00	
day 5-8	S1 (n)	3	1	0.25	0.90
	S2a, 2b, 3 (n)	0	6	0.00	
day 6-9	S1 (n)	0	3	1.00	0.67
	S2a, 2b, 3 (n)	0	6	0.00	
day 7-10	S1 (n)	0	3	1.00	0.63
	S2a, 2b, 3 (n)	0	5	0.00	
day 8-11	S1 (n)	0	4	1.00	0.64
	S2a, 2b, 3 (n)	0	7	0.00	
day 9-12	S1 (n)	0	4	1.00	0.60
	S2a, 2b, 3 (n)	0	6	0.00	

**Table 4:** The accuracy of the model to distinguish severity groups 2b and 3 from severity groups 1 and 2a in the cases of breakthrough infection.

A. Clinical data alone					
Day	True Severity	Estimated S1 (n)	estimate S2a, 2b, 3 (n)	Error Rate	Accuracy
day 4-7	S1, 2a (n)	6	0	0.00	0.67
	S2b, 3 (n)	3	0	1.00	
day 5-8	S1, 2a (n)	4	1	0.20	0.60
	S2b, 3 (n)	3	2	0.60	
day 6-9	S1, 2a (n)	3	1	0.25	0.56
	S2b, 3 (n)	3	2	0.60	
day 7-10	S1, 2a (n)	1	2	0.67	0.50
	S2b, 3 (n)	2	3	0.40	
day 8-11	S1, 2a (n)	3	2	0.40	0.45
	S2b, 3 (n)	4	2	0.67	
day 9-12	S1, 2a (n)	3	2	0.40	0.60
	S2b, 3 (n)	2	3	0.40	
**B. Clinical data + antibody data**					
**Day**	**True Severity**	**Estimated S1 (n)**	**Estimate S2a, 2b, 3 (n)**	**Error Rate (%)**	**Accuracy**
day 4-7	S1, 2a (n)	6	0	0.00	0.67
	S2b, 3 (n)	3	0	1.00	
day 5-8	S1, 2a (n)	5	0	0.00	0.50
	S2b, 3 (n)	5	0	1.00	
day 6-9	S1, 2a (n)	4	0	0.00	0.44
	S2b, 3 (n)	5	0	1.00	
day 7-10	S1, 2a (n)	3	0	0.00	0.38
	S2b, 3 (n)	5	0	1.00	
day 8-11	S1, 2a (n)	5	0	0.00	0.45
	S2b, 3 (n)	6	0	1.00	
day 9-12	S1, 2a (n)	3	2	0.40	0.50
	S2b, 3 (n)	3	2	0.60	

Considering that the antibodies against S1 and RBD should be especially modulated by vaccination, we further constructed the models for the prediction of maximum severity, using only the antibody data on IgM(N), IgG(N), and IgA(N). However, as shown in **Supplemental Figure S10** and **Supplemental Tables S6**, **S7**, in both models, the addition of the data on only IgM(N), IgG(N), and IgA(N) did not improve the accuracy of the models for predicting the maximum severity in the cases of breakthrough infections. The workflow to predict the maximum severity which represents one of tree estimators is shown in **Supplemental Figure S11** and the feature importance in the models is described in **Supplemental Figure S12**.

## Discussion

Numerous studies have demonstrated that the antibody responses to SARS-CoV-2 are associated with the clinical disease severity, however, the timing in the clinical course at which the associations are observed and the types of antibody responses that are associated with the disease maximum severity remain uncertain at present, as described in the *Introduction* section. Moreover, the clinical usefulness of antibody testing also needed to be demonstrated. The underlying issues for these limitations are that few studies have measured the serum titers of IgM, IgG, and IgA against the S protein, RBD, and N protein serially at short intervals span. In the present study, we serially measured nine types of antibodies simultaneously in samples obtained from COVID-19 patients, especially in the early phase of the disease, when determination of the disease maximum severity is clinically important. In addition, in this study, we subdivided COVID-19 patients of the disease severity group of 2, who require oxygen supplementation, but not mechanical respiratory support, into two groups: severity group 2a, that required supplemental oxygen at relatively low flow rates (under 4 L/min *via* a nasal cannula) and severity group 2b, who required oxygen supplementation at relatively high flow rates.

As shown in **Figures 2** and **3**, the antibody titers in the COVID-19 patients increased more rapidly in patients with more severe disease. Many studies conducted to date have failed to demonstrate associations between the antibody responses within 7 days of the disease onset and the disease severity (8–10, 16, 25, 27); this could be due to the limited number of samples analyzed or the analysis including the cumulative antibody titers from day 0 to day 7, although a few studies suggested early elevation, not reaching statistical significance, of IgG and IgA within one week from the onset in patients with more severe disease (12, 13). To the best of our knowledge, this is the first study to demonstrate elevation of various antibody types within one week from symptom onset in patients with COVID-19. In the early phase of the disease, within one week of the symptom onset, the titers of all the antibody types described above, except IgM(N), were higher in COVID-19 patients belonging to severity group 2b or 3, which suggested the possible usefulness of antibody testing to identify the subgroup of patients who would require oxygen supplementation at high flow rates. Moreover, although no significant difference was observed, the titers of IgG(S1) and IgG(RBD), which are considered as neutralizing antibodies, tended to be higher in severity group 2b than in severity group 3. This result might suggest that the requirement of mechanical respiratory support could be avoided in patients who can raise neutralizing antibodies sufficiently quickly, as also suggested by a previous study (46). Consistent with this hypothesis, IgG(S1/N) and IgG(RBD/N), as well as IgM(S1/N) and IgM(RBD/N) were higher in severity group 2b than in severity group 3 (**Supplemental Figure S2**).

To validate the clinical usefulness of antibody testing for predicting the maximum severity of COVID-19, we adopted a machine learning approach to establish analytical models. As expected from the associations between the antibody classes and the disease maximum severity, addition of the antibody data improved the ability for predicting severity groups 2b and 3, but not for predicting severity groups of 2a and over (**Tables S1**, **2** and **Figure 4**). In clinical practice, subjects of severity group 2a require hospitalization, while subjects of severity group 2b further require admission to the intensive care unit. Considering this situation, we believe that antibody testing will help physicians triage patients with COVID-19, especially identify patients who require admission to hospitals that are adequately equipped to deal with severe disease. In other words, antibody testing is expected to reduce the risk of underestimating the severity of COVID-19.

The limitations of the present study were 1) that the study was retrospective in nature, and 2) that the effects of mutations of SARS-CoV-2 were not taken into account, since the immune responses would be expected to be influenced by the strain of SARS-CoV-2. However, the samples for this study were collected from April 2020 to January 2021, when the N501Y, E484Q, and L452R variants were not yet prevalent in Japan, suggesting that the results of the present study would not have been confounded by the virus variant types. Nonetheless, a further prospective study considering the types of SARS-CoV-2 strains is needed to validate these findings.

Although the present study had been conducted when the vaccination had not prevailed in Japan, now the vaccination has prevailed and breakthrough infections are clinical concerns in many countries. We investigated the possible application of the models constructed with the data obtained from non-vaccinated COVID-19 subjects to the cases of breakthrough infections. As shown in **Tables 3** and **4**, the accuracy of the constructed models to predict the maximum severity of COVID-19 was not so high in the cases of breakthrough infections. Even when we used the antibody data on only IgM(N), IgG(N), and IgA(N), which was obtained from non-vaccinated subjects, the antibody data did not obviously improve the ability to predict the maximum severity in the cases of breakthrough infections (**Supplemental Tables S6**, **S7**). However, considering that the antibody data surely improved the ability of the models constructed using a machine learning technique to distinguish severity groups 2b and 3 from severity groups 1 and 2a and that the accuracy of the predicting models for severity groups 2b and 3, which was constructed only with clinical data, was relatively low in the cases of breakthrough infections (**Table 4A**), we expect that further studies with the antibody data in the cases of breakthrough infections will construct more proper models for the cases of breakthrough infections and also help physicians to triage patients who had taken vaccination.

In summary, the present study is the first study to clearly show that the titers of IgM, IgG and IgA against the S protein, RBD, and N protein increased rapidly according to the maximum severity of COVID-19, especially in those who required supplemental oxygen at high flow rates. Thus, antibody testing may be expected to help physicians in identifying non-vaccinated COVID-19 subjects who need admission to an intensive care unit.

## Data Availability Statement

The original contributions presented in the study are included in the article/**Supplementary Material**. Further inquiries can be directed to the corresponding author.

## Ethics Statement

The studies involving human participants were reviewed and approved by The University of Tokyo Medical Research Center Ethics Committee. Written informed consent to participate in this study was provided by the participants’ legal guardian/next of kin.

## Author Contributions

MK participated in the study design, experiments, and data analysis, and drafted the initial manuscript. HO participated in the data analysis and visualization. RY and YN participated in the experiments. CQ, FX, FH, LZ, YYu, YK, and JO developed the antibody measurement system. DJ, KO, KM, and TK participated in the discussion and helped in drafting the manuscript. YYa conceived the study, coordinated the study design, and helped in drafting the manuscript. All the authors have read and approved the final manuscript.

## Funding

This work was supported by Research Grants in the Natural Sciences from the Mitsubishi Foundation.

## Conflict of Interest

The present study was a collaborative research project among The University of Tokyo, Shenzhen YHLO Biotech Co., Ltd, and Medical & Biological Laboratories Co., Ltd. FX, FH, LZ, and YYu are employees of Shenzhen YHLO Biotech Co., Ltd and YK, JO, and HO are employees of Medical & Biological Laboratories Co., Ltd.

The remaining authors declare that the research was conducted in the absence of any commercial or financial relationships that could be construed as a potential conflict of interest.

## Publisher’s Note

All claims expressed in this article are solely those of the authors and do not necessarily represent those of their affiliated organizations, or those of the publisher, the editors and the reviewers. Any product that may be evaluated in this article, or claim that may be made by its manufacturer, is not guaranteed or endorsed by the publisher.

## References

[1] Ko JY Danielson ML Town M Derado G Greenlund KJ Kirley PD. Risk Factors for Coronavirus Disease 2019 (COVID-19)-Associated Hospitalization: COVID-19-Associated Hospitalization Surveillance Network and Behavioral Risk Factor Surveillance System. Clin Infect Dis (2021) 72:e695–703. doi: 10.1101/2020.07.27.20161810 PMC7543371 32945846

[2] Thakur B Dubey P Benitez J Torres JP Reddy S Shokar N. A Systematic Review and Meta-Analysis of Geographic Differences in Comorbidities and Associated Severity and Mortality Among Individuals With COVID-19. Sci Rep (2021) 11:8562. doi: 10.1038/s41598-021-88130-w 33879826 PMC8058064

[3] Broman N Rantasarkka K Feuth T Valtonen M Waris M Hohenthal U. IL-6 and Other Biomarkers as Predictors of Severity in COVID-19. Ann Med (2021) 53:410–2. doi: 10.1080/07853890.2020.1840621 PMC7935117 33305624

[4] Huang J Gao J Zhu W Feng R Liu Q Chen X. Indicators and Prediction Models for the Severity of Covid-19. Int J Clin Pract (2021) 75:e14571. doi: 10.1111/ijcp.14571 34170611 PMC8420422

[5] Gozalbo-Rovira R Gimenez E Latorre V Frances-Gomez C Albert E Buesa J. SARS-CoV-2 Antibodies, Serum Inflammatory Biomarkers and Clinical Severity of Hospitalized COVID-19 Patients. J Clin Virol (2020) 131:104611. doi: 10.1016/j.jcv.2020.104611 32882666 PMC7459327

[6] Kong WH Zhao R Zhou JB Wang F Kong DG Sun JB. Serologic Response to SARS-CoV-2 in COVID-19 Patients With Different Severity. Virol Sin (2020) 35:752–7. doi: 10.1007/s12250-020-00270-x PMC7376096 32705575

[7] Phipps WS Sorelle JA Li QZ Mahimainathan L Araj E Markantonis J. SARS-CoV-2 Antibody Responses Do Not Predict COVID-19 Disease Severity. Am J Clin Pathol (2020) 154:459–65. doi: 10.1093/ajcp/aqaa123 PMC7454292 32666092

[8] Liu L To KK Chan KH Wong YC Zhou R Kwan KY. High Neutralizing Antibody Titer in Intensive Care Unit Patients With COVID-19. Emerg Microbes Infect (2020) 9:1664–70. doi: 10.1080/22221751.2020.1791738 PMC7473321 32618497

[9] Rijkers G Murk JL Wintermans B Van Looy B Van Den Berge M Veenemans J. Differences in Antibody Kinetics and Functionality Between Severe and Mild Severe Acute Respiratory Syndrome Coronavirus 2 Infections. J Infect Dis (2020) 222:1265–9. doi: 10.1093/infdis/jiaa463 PMC7454692 32726417

[10] Roltgen K Powell AE Wirz OF Stevens BA Hogan CA Najeeb J. Defining the Features and Duration of Antibody Responses to SARS-CoV-2 Infection Associated With Disease Severity and Outcome. Sci Immunol (2020) 5:eabe0240. doi: 10.1126/sciimmunol.abe0240 33288645 PMC7857392

[11] Wang Y Zhang L Sang L Ye F Ruan S Zhong B. Kinetics of Viral Load and Antibody Response in Relation to COVID-19 Severity. J Clin Invest (2020) 130:5235–44. doi: 10.1172/JCI138759 PMC7524490 32634129

[12] Blackberg A Fernstrom N Sarbrant E Rasmussen M Sunnerhagen T. Antibody Kinetics and Clinical Course of COVID-19 a Prospective Observational Study. PloS One (2021) 16:e0248918. doi: 10.1371/journal.pone.0248918 33750984 PMC7984607

[13] Cervia C Nilsson J Zurbuchen Y Valaperti A Schreiner J Wolfensberger A. Systemic and Mucosal Antibody Responses Specific to SARS-CoV-2 During Mild Versus Severe COVID-19. J Allergy Clin Immunol (2021) 147:545–57.e549. doi: 10.1016/j.jaci.2020.10.040 33221383 PMC7677074

[14] De Donno A Lobreglio G Panico A Grassi T Bagordo F Bozzetti MP. IgM and IgG Profiles Reveal Peculiar Features of Humoral Immunity Response to SARS-CoV-2 Infection. Int J Environ Res Public Health (2021) 18:1318. doi: 10.3390/ijerph18031318 33535692 PMC7908175

[15] Fu Y Li Y Guo E He L Liu J Yang B. Dynamics and Correlation Among Viral Positivity, Seroconversion, and Disease Severity in COVID-19 : A Retrospective Study. Ann Intern Med (2021) 174:453–61. doi: 10.7326/M20-3337 PMC7745119 33284684

[16] Kutsuna S Asai Y Matsunaga A Kinoshita N Terada M Miyazato Y. Factors Associated With Anti-SARS-CoV-2 IgG Antibody Production in Patients Convalescing From COVID-19. J Infect Chemother (2021) 27:808–13. doi: 10.1016/j.jiac.2021.01.006 PMC7836855 33531292

[17] Legros V Denolly S Vogrig M Boson B Siret E Rigaill J. A Longitudinal Study of SARS-CoV-2-Infected Patients Reveals a High Correlation Between Neutralizing Antibodies and COVID-19 Severity. Cell Mol Immunol (2021) 18:318–27. doi: 10.1038/s41423-020-00588-2 PMC7786875 33408342

[18] Patil HP Rane PS Shrivastava S Palkar S Lalwani S Mishra AC. Antibody (IgA, IgG, and IgG Subtype) Responses to SARS-CoV-2 in Severe and Nonsevere COVID-19 Patients. Viral Immunol (2021) 34:201–9. doi: 10.1089/vim.2020.0321 33656935

[19] Ravichandran S Lee Y Grubbs G Coyle EM Klenow L Akasaka O. Longitudinal Antibody Repertoire in "Mild" Versus "Severe" COVID-19 Patients Reveals Immune Markers Associated With Disease Severity and Resolution. Sci Adv (2021) 7:eabf2467. doi: 10.1126/sciadv.abf2467 33674317 PMC7935365

[20] Shrivastava S Palkar S Shah J Rane P Lalwani S Mishra AC. Early and High SARS-CoV-2 Neutralizing Antibodies Are Associated With Severity in COVID-19 Patients From India. Am J Trop Med Hyg (2021) 105:401–6. doi: 10.4269/ajtmh.21-0014 PMC8437163 34138748

[21] Chen W Zhang J Qin X Wang W Xu M Wang LF. SARS-CoV-2 Neutralizing Antibody Levels are Correlated With Severity of COVID-19 Pneumonia. BioMed Pharmacother (2020) 130:110629. doi: 10.1016/j.biopha.2020.110629 33406577 PMC7425713

[22] Crawford KHD Dingens AS Eguia R Wolf CR Wilcox N Logue JK. Dynamics of Neutralizing Antibody Titers in the Months After SARS-CoV-2 Infection. J Infect Dis (2021) 223:197–205. doi: 10.1093/infdis/jiaa618 33535236 PMC7543487

[23] Liu X Zheng X Liu B Wu M Zhang Z Zhang G. Serum IgM Against SARS-CoV-2 Correlates With in-Hospital Mortality in Severe/Critical Patients With COVID-19 in Wuhan, China. Aging (Albany NY) (2020) 12:12432–40. doi: 10.18632/aging.103417 PMC7377873 32628642

[24] Marklund E Leach S Axelsson H Nystrom K Norder H Bemark M. Serum-IgG Responses to SARS-CoV-2 After Mild and Severe COVID-19 Infection and Analysis of IgG non-Responders. PloS One (2020) 15:e0241104. doi: 10.1371/journal.pone.0241104 33085715 PMC7577439

[25] Ren L Zhang L Chang Wang J Hu Y Chen H. The Kinetics of Humoral Response and its Relationship With the Disease Severity in COVID-19. Commun Biol (2020) 3:780. doi: 10.1038/s42003-020-01526-8 33311543 PMC7733479

[26] Schlickeiser S Schwarz T Steiner S Wittke K Al Besher N Meyer O. Disease Severity, Fever, Age, and Sex Correlate With SARS-CoV-2 Neutralizing Antibody Responses. Front Immunol (2020) 11:628971. doi: 10.3389/fimmu.2020.628971 33584731 PMC7878374

[27] Zhao J Yuan Q Wang H Liu W Liao X Su Y. Antibody Responses to SARS-CoV-2 in Patients With Novel Coronavirus Disease 2019. Clin Infect Dis (2020) 71:2027–34. doi: 10.1093/cid/ciaa344 PMC7184337 32221519

[28] Chia WN Zhu F Ong SWX Young BE Fong SW Le Bert N. Dynamics of SARS-CoV-2 Neutralising Antibody Responses and Duration of Immunity: A Longitudinal Study. Lancet Microbe (2021) 2:e240–9. doi: 10.1016/S2666-5247(21)00025-2 PMC7987301 33778792

[29] Garcia-Beltran WF Lam EC Astudillo MG Yang D Miller TE Feldman J. COVID-19-Neutralizing Antibodies Predict Disease Severity and Survival. Cell (2021) 184:476–88.e411. doi: 10.1016/j.cell.2020.12.015 33412089 PMC7837114

[30] Gerhards C Thiaucourt M Kittel M Becker C Ast V Hetjens M. Longitudinal Assessment of Anti-SARS-CoV-2 Antibody Dynamics and Clinical Features Following Convalescence From a COVID-19 Infection. Int J Infect Dis (2021) 107:221–7. doi: 10.1016/j.ijid.2021.04.080 PMC8080496 33932604

[31] Xu X Nie S Wang Y Long Q Zhu H Zhang X. Dynamics of Neutralizing Antibody Responses to SARS-CoV-2 in Patients With COVID-19: An Observational Study. Signal Transduct Target Ther (2021) 6:197. doi: 10.1038/s41392-021-00611-6 34006847 PMC8129700

[32] Yates JL Ehrbar DJ Hunt DT Girardin RC Dupuis AP 2nd Payne AF. Serological Analysis Reveals an Imbalanced IgG Subclass Composition Associated With COVID-19 Disease Severity. Cell Rep Med (2021) 2:100329. doi: 10.1016/j.xcrm.2021.100329 34151306 PMC8205277

[33] Batra M Tian R Zhang C Clarence E Sacher CS Miranda JN. Role of IgG Against N-Protein of SARS-CoV2 in COVID19 Clinical Outcomes. Sci Rep (2021) 11:3455. doi: 10.1038/s41598-021-83108-0 33568776 PMC7875990

[34] Trinite B Tarres-Freixas F Rodon J Pradenas E Urrea V Marfil S. SARS-CoV-2 Infection Elicits a Rapid Neutralizing Antibody Response That Correlates With Disease Severity. Sci Rep (2021) 11:2608. doi: 10.1038/s41598-021-81862-9 33510275 PMC7843981

[35] Seow J Graham C Merrick B Acors S Pickering S Steel KJA. Longitudinal Observation and Decline of Neutralizing Antibody Responses in the Three Months Following SARS-CoV-2 Infection in Humans. Nat Microbiol (2020) 5:1598–607. doi: 10.1038/s41564-020-00813-8 PMC7610833 33106674

[36] Wang P Liu L Nair MS Yin MT Luo Y Wang Q. SARS-CoV-2 Neutralizing Antibody Responses are More Robust in Patients With Severe Disease. Emerg Microbes Infect (2020) 9:2091–3. doi: 10.1080/22221751.2020.1823890 PMC7534308 32930052

[37] Pierce CA Preston-Hurlburt P Dai Y Aschner CB Cheshenko N Galen B. Immune Responses to SARS-CoV-2 Infection in Hospitalized Pediatric and Adult Patients. Sci Transl Med (2020) 12:eabd5487. doi: 10.1126/scitranslmed.abd5487 32958614 PMC7658796

[38] Nakano Y Kurano M Morita Y Shimura T Yokoyama R Qian C. Time Course of the Sensitivity and Specificity of Anti-SARS-CoV-2 IgM and IgG Antibodies for Symptomatic COVID-19 in Japan. Sci Rep (2021) 11:2776. doi: 10.1038/s41598-021-82428-5 33531605 PMC7854735

[39] Qian C Zhou M Cheng F Lin X Gong Y Xie X. Development and Multicenter Performance Evaluation of Fully Automated SARS-CoV-2 IgM and IgG Immunoassays. Clin Chem Lab Med (2020) 58:1601–7. doi: 10.1515/cclm-2020-0548 32609640

[40] Brunner E Munzel U. The Nonparametric Behrens-Fisher Problem: Asymptotic Theory and a Small-Sample Approximation. Biom J (2000) 42:17–25. doi: 10.1002/(SICI)1521-4036(200001)42:1

[41] Royston JP. An Extension of Shapiro and Wilk-W Test for Normality to Large Samples. J R Stat Soc Ser C Appl Stat (1982) 31:115–24.doi: 10.2307/2347973

[42] Chen TQ Guestrin C. XGBoost: A Scalable Tree Boosting System. In: Kdd’16: Proceedings of the 22nd Acm Sigkdd International Conference on Knowledge Discovery and Data Mining. New York, NY, USA: ACM (2016). p. 785–94.

[43] Wang F Huang S Gao R Zhou Y Lai C Li Z. Initial Whole-Genome Sequencing and Analysis of the Host Genetic Contribution to COVID-19 Severity and Susceptibility. Cell Discov (2020) 6:83. doi: 10.1038/s41421-020-00231-4 33298875 PMC7653987

[44] Guan X Zhang B Fu M Li M Yuan X Zhu Y. Clinical and Inflammatory Features Based Machine Learning Model for Fatal Risk Prediction of Hospitalized COVID-19 Patients: Results From a Retrospective Cohort Study. Ann Med (2021) 53:257–66. doi: 10.1080/07853890.2020.1868564 PMC7799376 33410720

[45] Sun C Bai Y Chen D He L Zhu J Ding X. Accurate Classification of COVID-19 Patients With Different Severity via Machine Learning. Clin Transl Med (2021) 11:e323. doi: 10.1002/ctm2.323 33784017 PMC7908044

[46] Lucas C Klein J Sundaram ME Liu F Wong P Silva J. Delayed Production of Neutralizing Antibodies Correlates With Fatal COVID-19. Nat Med (2021) 27:1178–86. doi: 10.1038/s41591-021-01355-0 PMC8785364 33953384

