# Supplementary material for: Measurement of SARS-CoV-2 Antibody Titers Improves the Prediction Accuracy of COVID-19 Maximum Severity by Machine Learning in Non-Vaccinated Patients
Source: Front Immunol. 2022 Jan 21;13:811952. doi: 10.3389/fimmu.2022.811952 (PMC8814445; doi:10.3389/fimmu.2022.811952)
Supplement: Supplementary file 1 [file DataSheet_1.pdf]

Supplemental Table S1. Characteristics of the subjects enrolled in this study.

	Severity group 1	Severity group 2a	Severity group 2b	Severity group 3
Age (mean ± S.D)	50 ± 19	63 ± 13*	63 ± 14*	64 ± 14*
Gender (male/female, n)	22 / 19	29 / 12	20 / 5	22 / 5
Diabetes mellitus (%)	24	29	44	41
Hypertension (%)	29	54	52	56
Current smoking (%)	10	22	20	22
Drugs used:				
Favipiravir (%)	34	51	32	52
Nafamostat (%)**	12	20	24	52
Remdesivir (%)**	5	41	64	33
Steroids (%)**	12	54	76	85

Age is represented as the median ± S.D. The differences in age among the three severity classes of COVID-19 were assessed using an independent Kruskal-Wallis test, followed by the Games Howell test for post hoc analysis. * P < 0.01 vs. severity 1. The differences in the sex distribution, frequency of complications, and rate of use of specific drugs among the three patient groups classified according to the severity of COVID-19 were assessed by the Chi square test. ** P < 0.01.

Supplemental Table S2. Summary of the training and validation datasets.

Models	Number of samples (%)			
	Training set		Validation set	
	Low severity group	High severity group	Low severity group	High severity group
Day 4-7 S1 vs. S2a, 2b, 3	23 (35)	42 (65)	9 (33)	18 (67)
Day 4-7 S1, 2a vs. S2b, 3	52 (83)	12 (18)	22 (81)	5 (19)
Day 5-8 S1 vs. S2a, 2b, 3	25 (30)	58 (70)	10 (29)	24 (71)
Day 5-8 S1, 2a vs. S2b, 3	62 (77)	19 (23)	27 (77)	8 (23)
Day 6-9 S1 vs. S2a, 2b, 3	27 (29)	67 (71)	11 (28)	28 (72)
Day 6-9 S1, 2a vs. S2b, 3	65 (69)	29 (31)	27 (69)	12 (31)
Day 7-10 S1 vs. S2a, 2b, 3	28 (26)	78 (74)	12 (27)	33 (73)
Day 7-10 S1, 2a vs. S2b, 3	70 (66)	37 (35)	29 (66)	15 (34)
Day 8-11 S1 vs. S2a, 2b, 3	27 (20)	105 (80)	12 (27)	33 (73)
Day 8-11 S1, 2a vs. S2b, 3	71 (57)	54 (43)	30 (58)	22 (42)
Day 9-12 S1 vs. S2a, 2b, 3	26 (20)	105 (80)	12 (21)	45 (79)
Day 9-12 S1, 2a vs. S2b, 3	67 (51)	64 (49)	29 (52)	27 (48)

Supplemental Table S3. Drawn optimal hyperparameter values for each model.

Best values for models		Hyperparameters			
		Max length	Min child weight	Colsample bytree	Subsample
Possible values		3, 5, 7, 9, 10, 11	0, 0.2, 0.4, 0.6, 0.8, 1	0, 0.3, 0.5, 0.7, 0.9, 1.0	0, 0.3, 0.5, 0.7, 0.9, 1.0
Day 4-7 S1 vs. S2a, 2b, 3	clinical	10	0	0.3	0.7
	clinical + antibody	10	0.4	0.7	0.9
Day 4-7 S1, 2a vs. S2b, 3	clinical	10	0	1.0	1.0
	clinical + antibody	10	0.4	0.3	0.5
Day 5-8 S1 vs. S2a, 2b, 3	clinical	10	0.4	0.3	0.5
	clinical + antibody	10	0.6	0.3	1.0
Day 5-8 S1, 2a vs. S2b, 3	clinical	10	0.4	0.5	0.7
	clinical + antibody	10	0.4	0.9	0.9
Day 6-9 S1 vs. S2a, 2b, 3	clinical	10	0.4	0.5	0.5
	clinical + antibody	10	0	0.9	0.3
Day 6-9 S1, 2a vs. S2b, 3	clinical	10	0	0.7	0.7
	clinical + antibody	10	0.8	1.0	0.3
Day 7-10 S1 vs. S2a, 2b, 3	clinical	10	0.8	0.3	0.3
	clinical + antibody	10	0.4	0.9	1.0
Day 7-10 S1, 2a vs. S2b, 3	clinical	10	0.2	0.7	0.5
	clinical + antibody	10	0.2	0.3	0.5
Day 8-11 S1 vs. S2a, 2b, 3	clinical	10	0.8	0.7	1.0
	clinical + antibody	10	0	1.0	0.7
Day 8-11 S1, 2a vs.	clinical	10	0	0.5	0.5

S2b, 3	clinical + antibody	10	0	0.5	0.7
Day 9-12 S1 vs. S2a, 2b, 3	clinical	10	0.8	0.3	0.5
	clinical + antibody	10	0.2	0.3	0.5
Day 9-12 S1, 2a vs. S2b, 3	clinical	10	0.2	0.7	0.5
	clinical + antibody	10	0.8	0.3	0.7

“clinical” indicates that variables of age, gender, diabetes mellitus, hypertension, current smoking, CRP, and D-Dimer were input into the models, and “clinical + antibody” indicates that the clinical information described above plus the antibody titers were input into the models.

Supplemental Table S4. The accuracy of the model to distinguish severity group 2a or over from severity group 1 in the validation set.

A. Clinical data alone

day	true severity	estimated S1 (n)	estimate S2a, 2b, 3 (n)	error rate	accuracy
day 1-6	S1 (n)	4	3	0.43	0.80
	S2a, 2b, 3 (n)	1	12	0.08	
day 7-12	S1 (n)	14	3	0.18	0.91
	S2a, 2b, 3 (n)	4	55	0.07	

B. Clinical data + antibody data

day	true severity	estimated S1 (n)	estimate S2a, 2b, 3 (n)	error rate (%)	accuracy
day 1-6	S1 (n)	5	2	0.29	0.85
	S2a, 2b, 3 (n)	1	12	0.08	
day 7-12	S1 (n)	10	7	0.41	0.83
	S2a, 2b, 3 (n)	6	53	0.10	

Supplemental Table S5. The accuracy of the model to distinguish severity groups 2b and 3 from severity groups 1 and 2a in the validation set.

A. Clinical data alone

day	true severity	estimated S1, 2a (n)	estimate S2b, 3 (n)	error rate (%)	accuracy
day 1-6	S1, 2a (n)	15	1	0.06	0.85
	S2b, 3 (n)	2	2	0.50	
day 7-12	S1, 2a (n)	37	7	0.16	0.76
	S2b, 3 (n)	11	21	0.34	

B. Clinical data + antibody data

day	true severity	estimated S1 (n)	estimate S2a, 2b, 3 (n)	error rate (%)	accuracy
day 1-6	S1, 2a (n)	15	1	0.06	0.90
	S2b, 3 (n)	1	3	0.25	
day 7-12	S1, 2a (n)	35	9	0.20	0.79
	S2b, 3 (n)	7	25	0.22	

Supplemental Table S6. The accuracy of the model to distinguish severity group 2a or over from severity group 1 in the validation set and the cases of breakthrough infections.

A. The validation set (Clinical data + antibody against N antigen)

day	true severity	estimated S1 (n)	estimate S2a, 2b, 3 (n)	error rate (%)	accuracy
day 4-7	S1 (n)	7	2	0.22	0.81
	S2a, 2b, 3 (n)	3	15	0.17	
day 5-8	S1 (n)	4	6	0.60	0.76
	S2a, 2b, 3 (n)	2	22	0.08	
day 6-9	S1 (n)	9	2	0.18	0.92
	S2a, 2b, 3 (n)	1	27	0.04	
day 7-10	S1 (n)	6	6	0.50	0.84
	S2a, 2b, 3 (n)	1	32	0.03	
day 8-11	S1 (n)	3	8	0.73	0.81
	S2a, 2b, 3 (n)	2	39	0.05	
day 9-12	S1 (n)	5	6	0.55	0.84
	S2a, 2b, 3 (n)	3	42	0.07	

B. The cases of breakthrough infections (Clinical data + antibodies against N antigen)

day	true severity	estimated S1 (n)	estimate S2a, 2b, 3 (n)	error rate (%)	accuracy
day 4-7	S1 (n)	4	1	0.20	0.78
	S2a, 2b, 3 (n)	1	3	0.25	
day 5-8	S1 (n)	3	1	0.25	0.90
	S2a, 2b, 3 (n)	0	6	0.00	
day 6-9	S1 (n)	1	2	0.67	0.75
	S2a, 2b, 3 (n)	0	6	0.00	
day 7-10	S1 (n)	1	2	0.67	0.75
	S2a, 2b, 3 (n)	0	5	0.00	
day 8-11	S1 (n)	0	4	1.00	0.64
	S2a, 2b, 3 (n)	0	7	0.00	
day 9-12	S1 (n)	0	4	1.00	0.60
	S2a, 2b, 3 (n)	0	6	0.00	

Supplemental Table S7. The accuracy of the model to distinguish severity groups 2b and 3 from severity groups 1 and 2a in the validation set and the cases of breakthrough infections.

A. The validation set (Clinical data + antibody against N antigen)

day	true severity	estimated S1 (n)	estimate S2a, 2b, 3 (n)	error rate (%)	accuracy
day 4-7	S1, 2a (n)	21	1	0.05	0.89
	S2b, 3 (n)	2	3	0.40	
day 5-8	S1, 2a (n)	24	3	0.11	0.80
	S2b, 3 (n)	4	4	0.50	
day 6-9	S1, 2a (n)	25	2	0.07	0.87
	S2b, 3 (n)	3	9	0.25	
day 7-10	S1, 2a (n)	26	3	0.10	0.75
	S2b, 3 (n)	8	7	0.53	
day 8-11	S1, 2a (n)	22	8	0.27	0.75
	S2b, 3 (n)	5	17	0.23	
day 9-12	S1, 2a (n)	24	5	0.17	0.77
	S2b, 3 (n)	8	19	0.30	

B. The cases of breakthrough infections (Clinical data + antibodies against N antigen)

day	true severity	estimated S1 (n)	estimate S2a, 2b, 3 (n)	error rate (%)	accuracy
day 4-7	S1, 2a (n)	6	0	0.00	0.67
	S2b, 3 (n)	3	0	1.00	
day 5-8	S1, 2a (n)	5	0	0.00	0.60
	S2b, 3 (n)	4	1	0.80	
day 6-9	S1, 2a (n)	4	0	0.00	0.56
	S2b, 3 (n)	4	1	0.80	
day 7-10	S1, 2a (n)	2	1	0.33	0.25
	S2b, 3 (n)	5	0	1.00	
day 8-11	S1, 2a (n)	3	2	0.40	0.36
	S2b, 3 (n)	5	1	0.83	
day 9-12	S1, 2a (n)	3	2	0.40	0.40
	S2b, 3 (n)	4	1	0.80	

Supplemental Figure S1

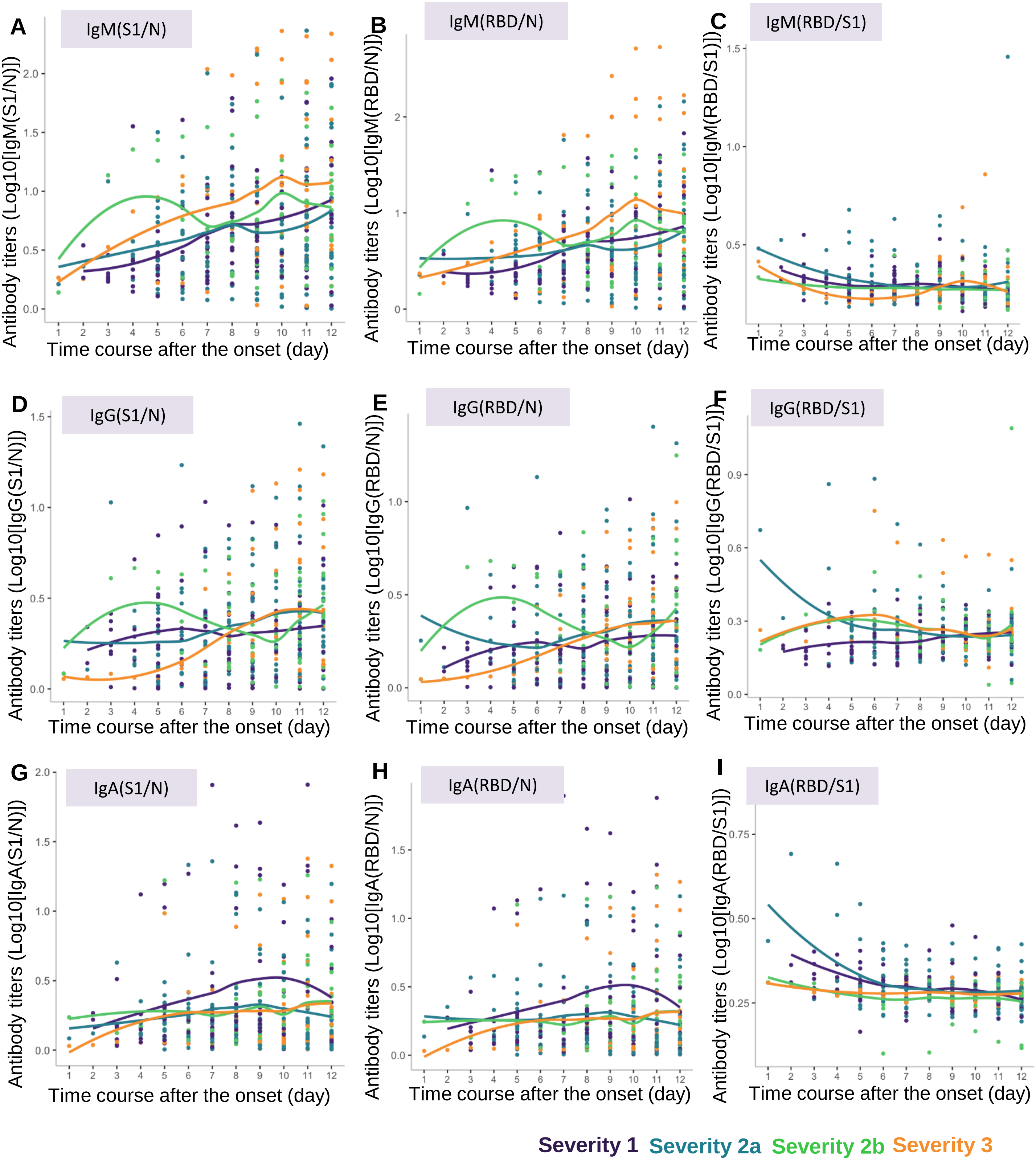

Supplemental Figure S1. Approximate curves for the antibody ratio kinetics in COVID-19 patients classified by the disease maximum severity.

Local polynomial regression curves were fitted to indicate the antibody ratios until day 12 after symptom onset in COVID-19 patients classified according to the disease severity. (A) IgM(S1/N), (B) IgM(RBD/N), (C) IgM(RBD/S1), (D) IgG(S1/N), (E) IgG(RBD/N), (F) IgG(RBD/S1), (G) IgA(S1/N), (H) IgA(RBD/N), (I) IgA(S1/RBD).

Supplemental Figure S2

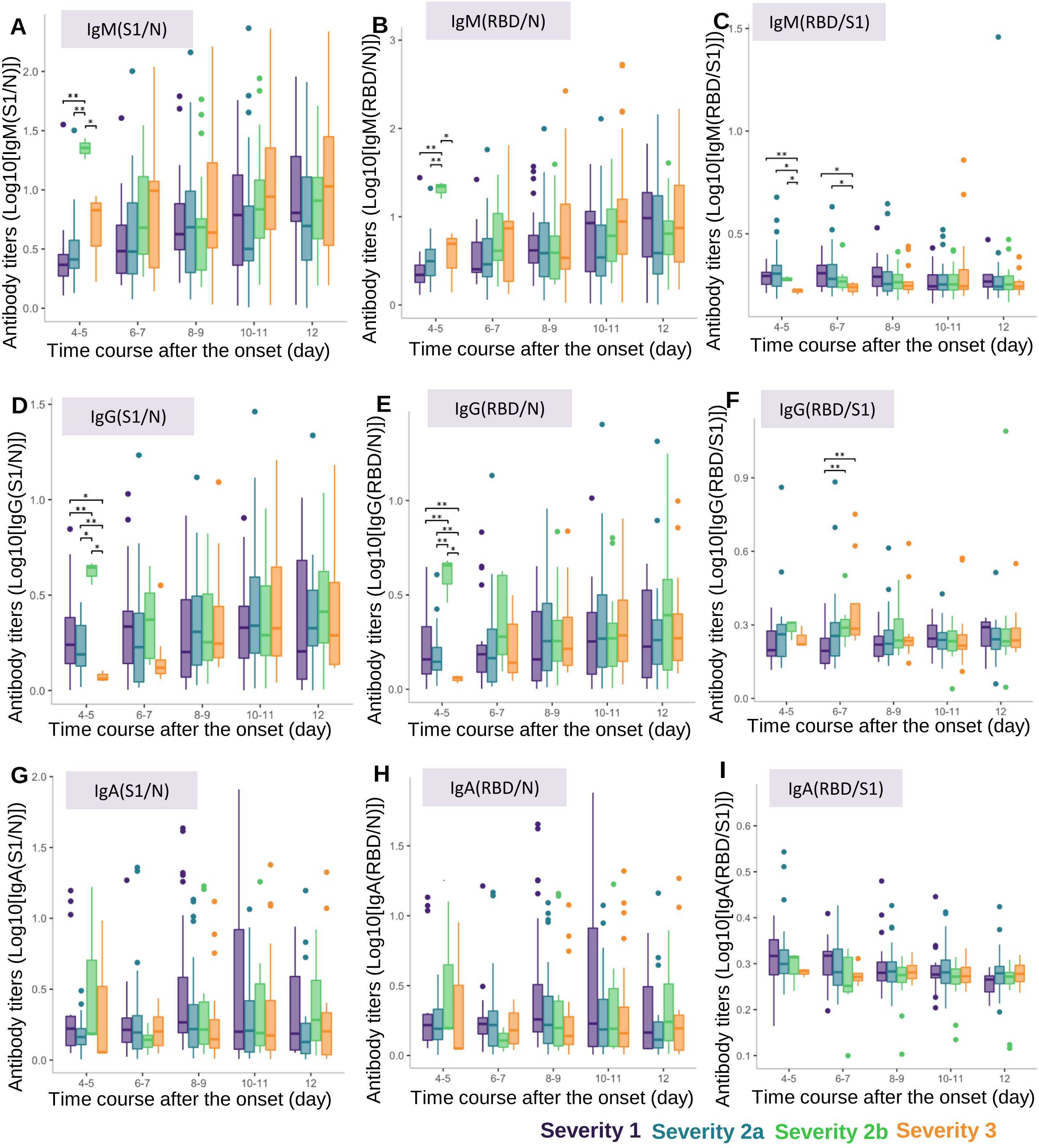

Supplemental Figure S2. The difference in antibody ratios among COVID-19 patients classified by the disease maximum severity.

We compared the various antibody ratios among patients with COVID-19 classified by the disease severity, as described in the *Material and Methods* section, on day 4-5, day 6-7, day 8-9, day 10-11, day 12 after symptom onset. $*P < 0.05$, $**P < 0.01$. The horizontal bar represents the median, the box bar represents the lower and upper quartiles, and the fine bar represents the minimum and maximum.

Supplemental Figure S3

Clinical data

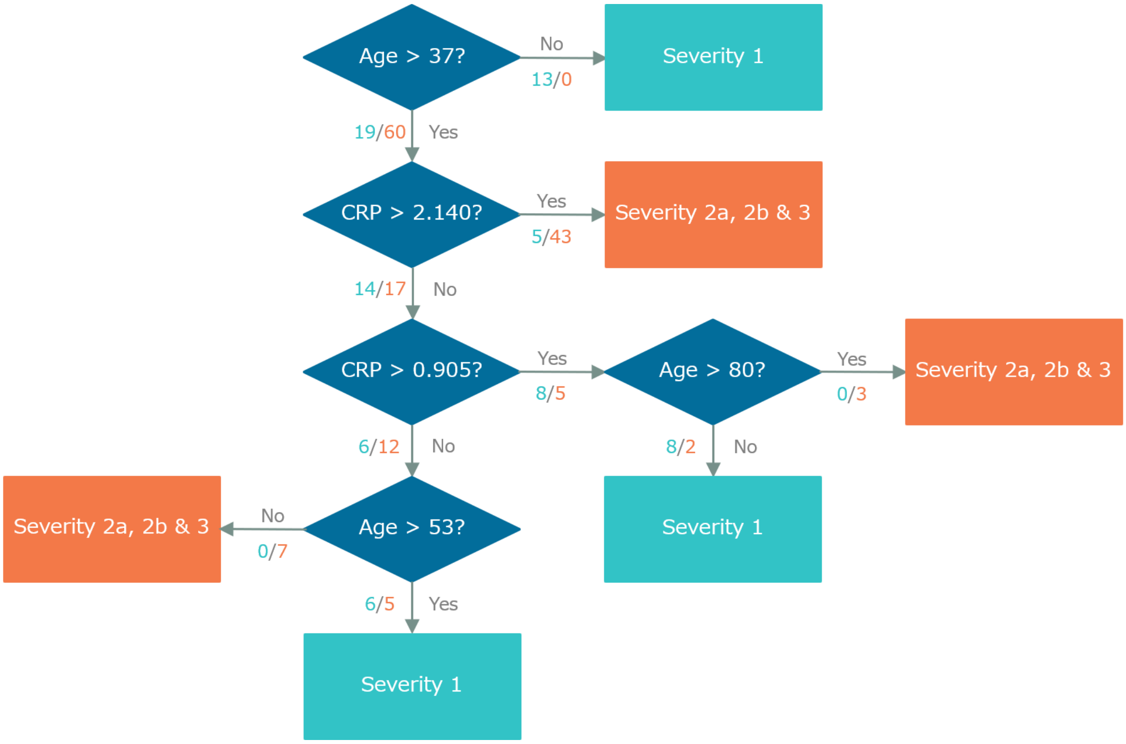

Clinical data + antibody data

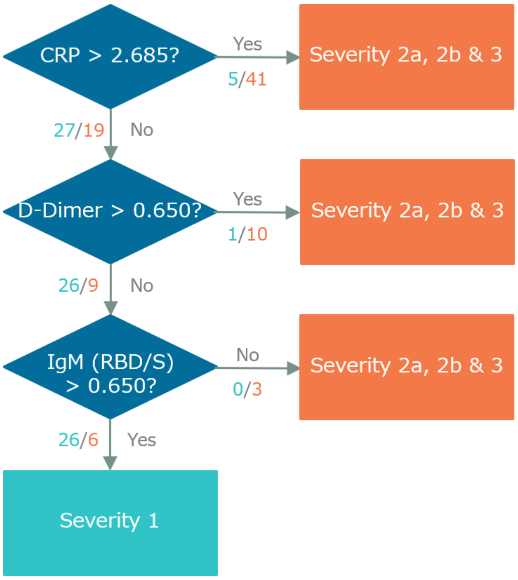

Supplemental Figure S3. The workflow to predict severity groups of 2a or over, which represents one of tree estimators in the optimum model, on day 4-7.

Supplemental Figure S4

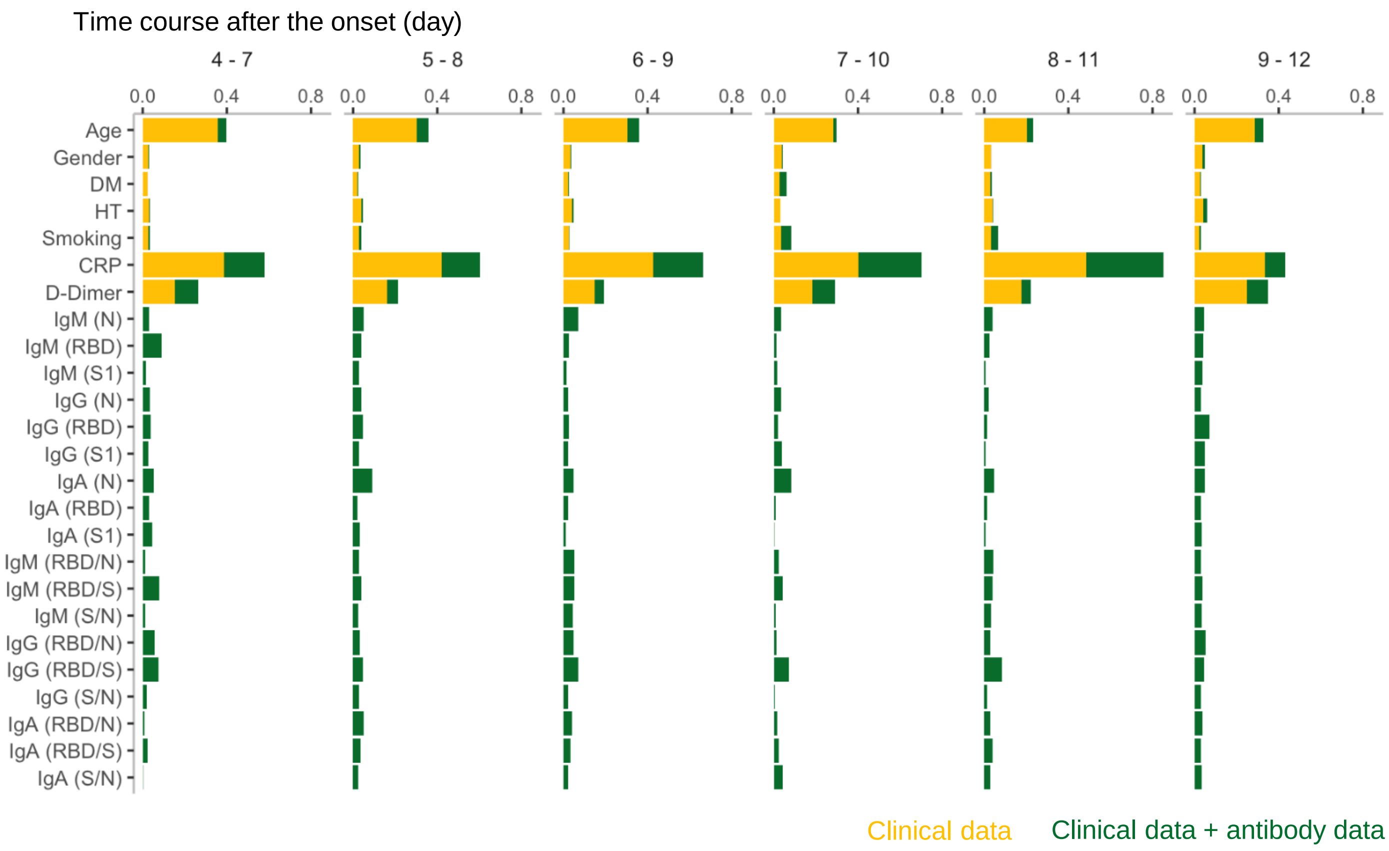

Supplemental Figure S4. The feature importance in the model constructed using a machine learning technique to distinguish severity groups of 2a or over and severity group 1.

Supplemental Figure S5

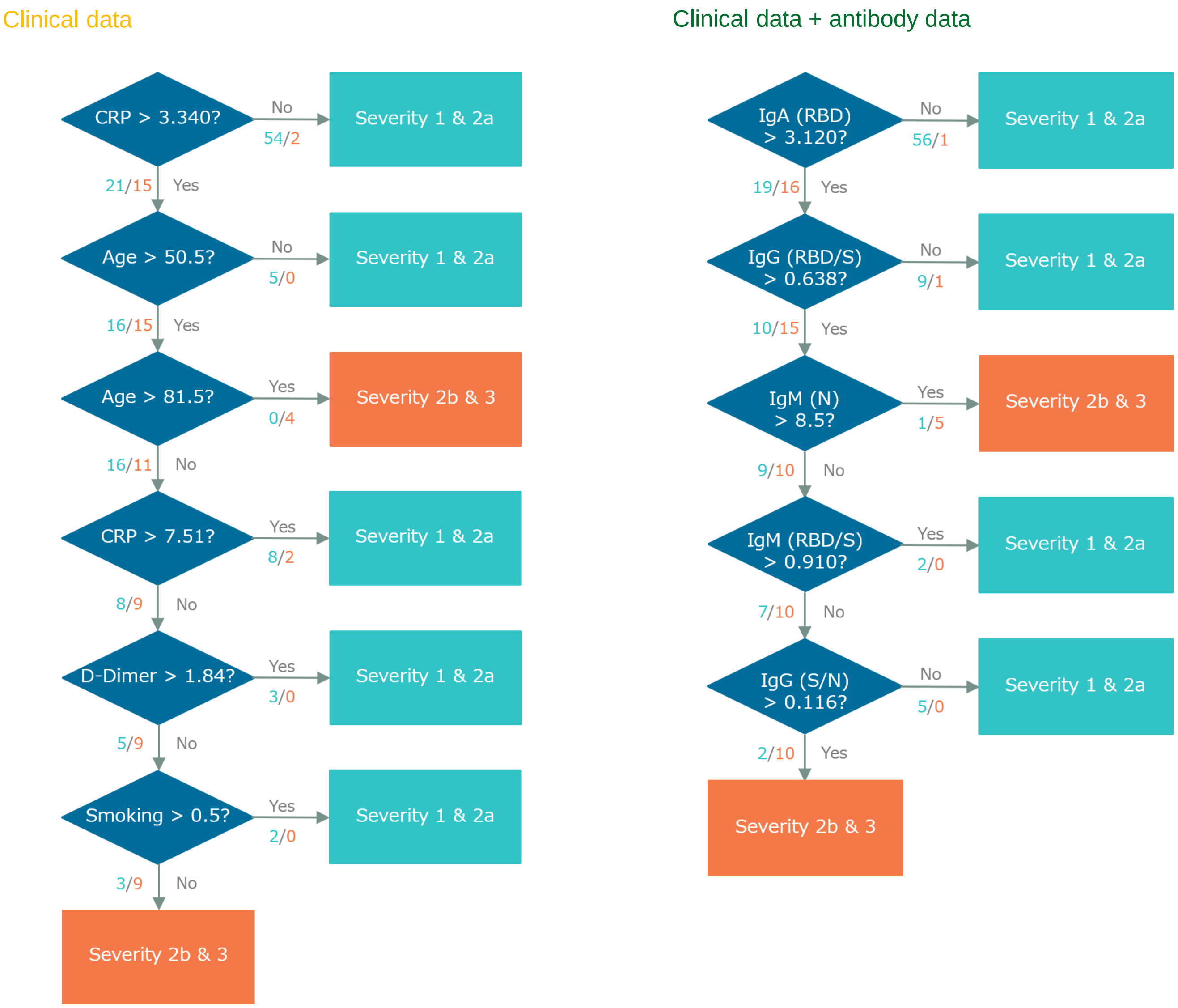

Supplemental Figure S5. The workflow to predict severity group 2b or 3, which represents one of the tree estimators in the optimum model on days 4-7.

Supplemental Figure S6

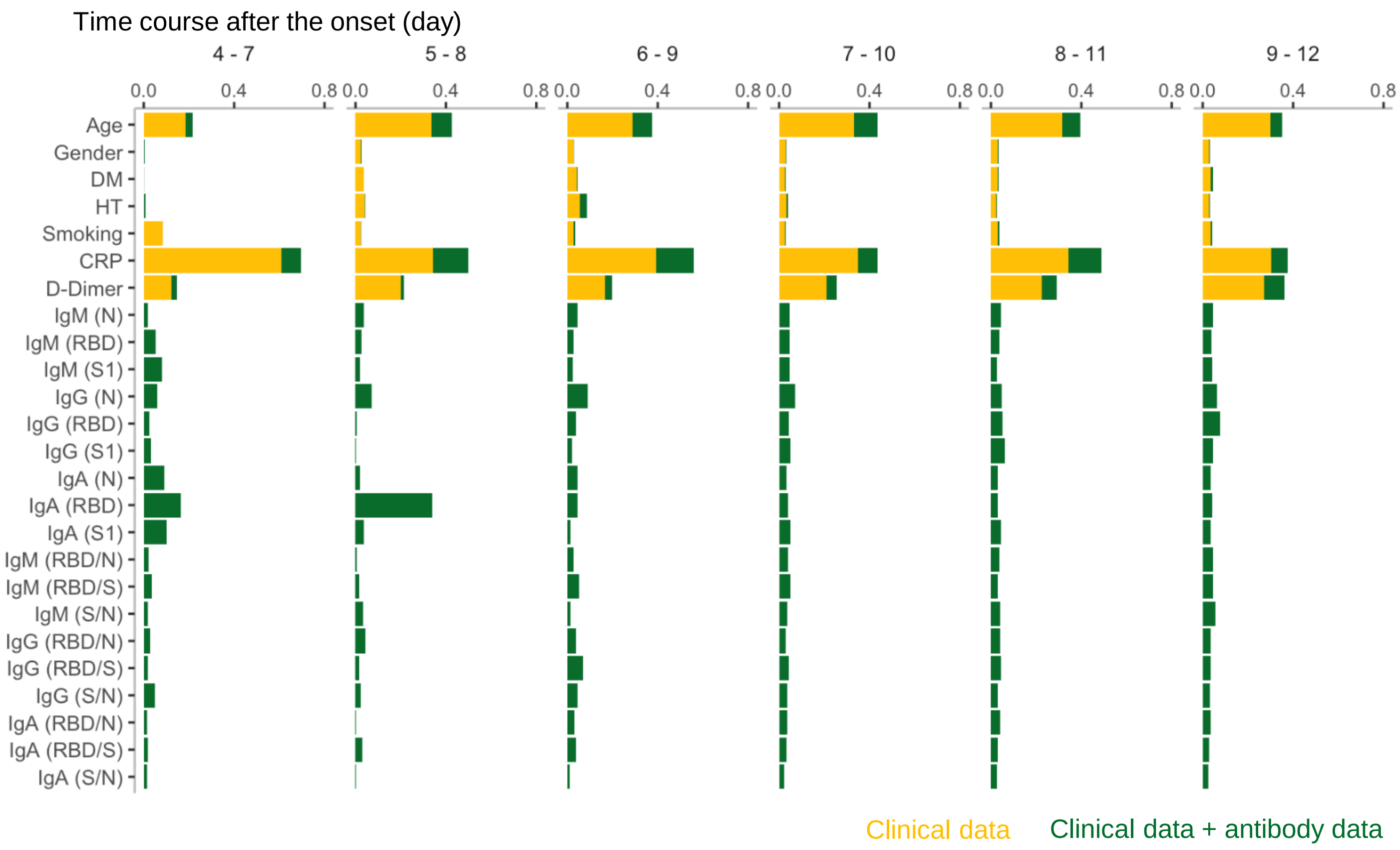

Supplemental Figure S6. The feature importance in the model constructed by a machine learning technique to distinguish severity groups 2b and 3 from severity groups 1 and 2a.

Supplemental Figure S7

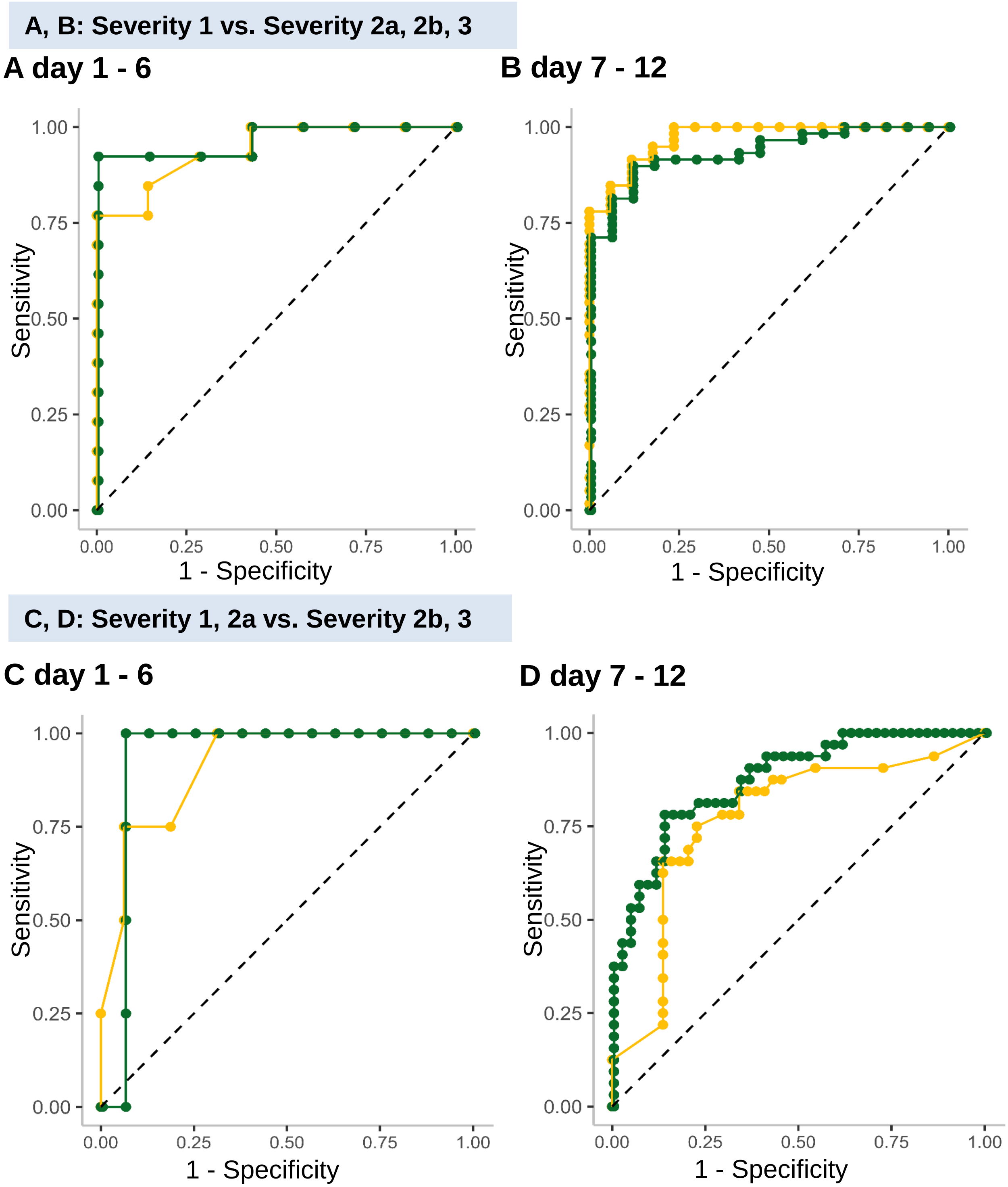

Supplemental Figure S7. ROC analyses of the analysis models constructed using a machine learning technique for predicting the maximum severity of COVID-19 when we sub-grouped the data on day 1-6 and day 7-12.

The ROCs of the analysis models constructed using a machine learning technique for predicting the COVID-19 severity, using the data obtained on day 1–6 (A, C), and day 7–12 (B, D), are shown. The models were constructed to distinguish severity groups 2a or over from severity group 1 (A, B) or distinguish severity groups 2b and 3 from severity groups 1 and 2a (C, D). The yellow curves represent the ROCs of the model constructed using clinical parameters and the green curves represent those of the model constructed using both clinical and antibody data.

Supplemental Figure S8

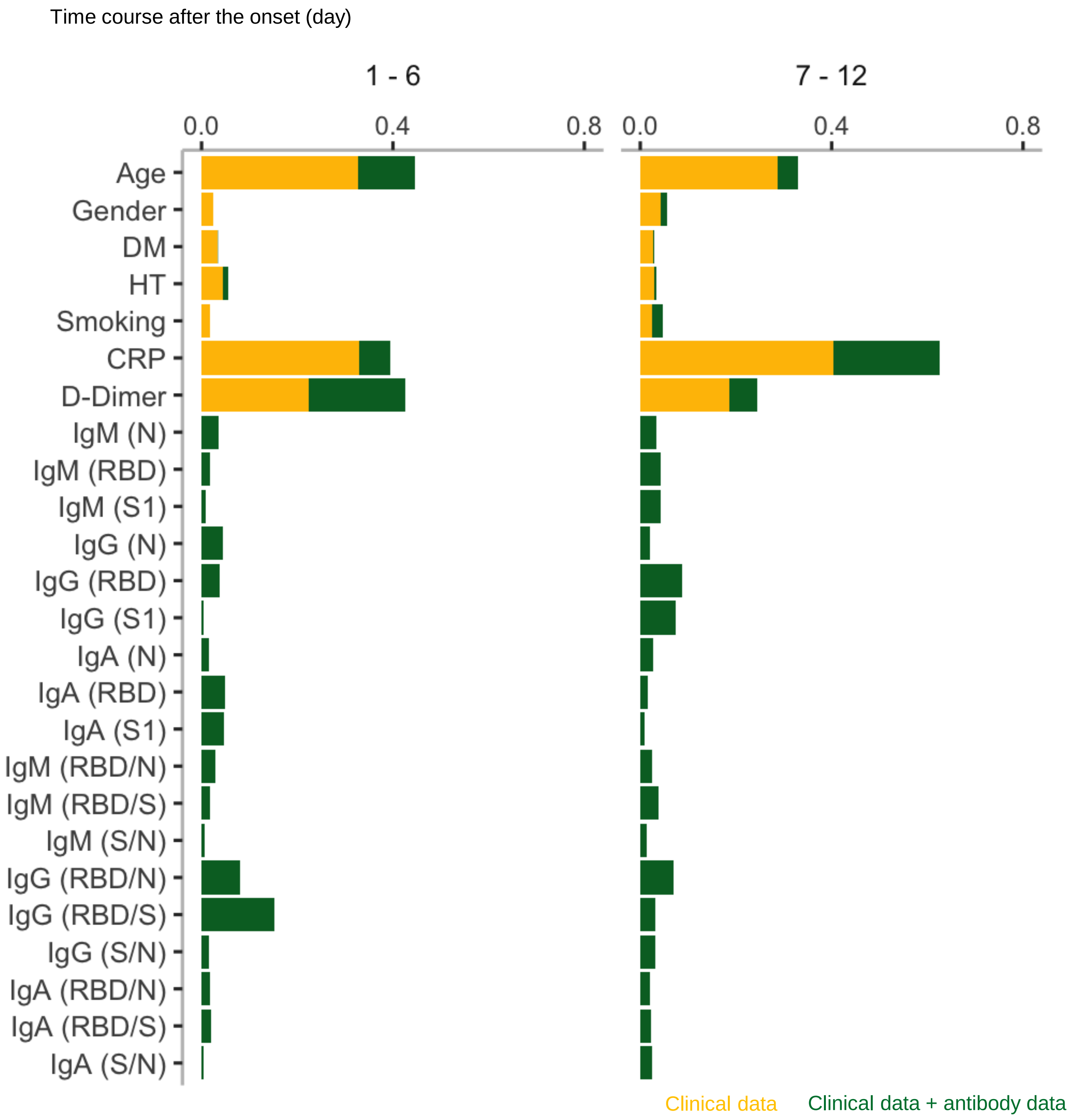

Supplemental Figure S8. The feature importance in the model constructed using a machine learning technique to distinguish severity groups of 2a or over and severity group 1, when we sub-grouped the data on day 1-6 and day7-12.

Supplemental Figure S9

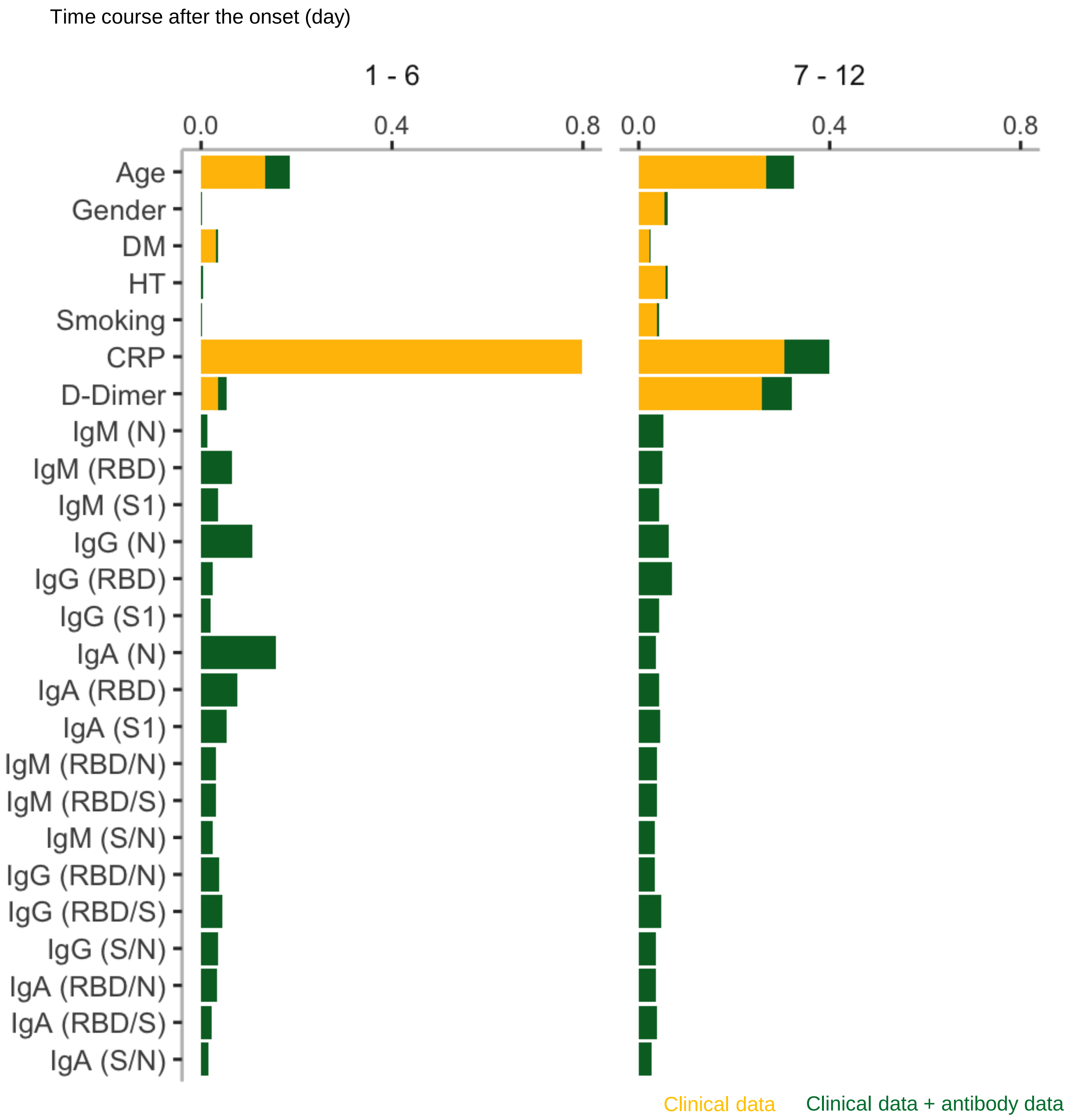

Supplemental Figure S9. The feature importance in the model constructed by a machine learning technique to distinguish severity groups 2b and 3 from severity groups 1 and 2a, when we sub-grouped the data on day 1-6 and day7-12.

Supplemental Figure S10

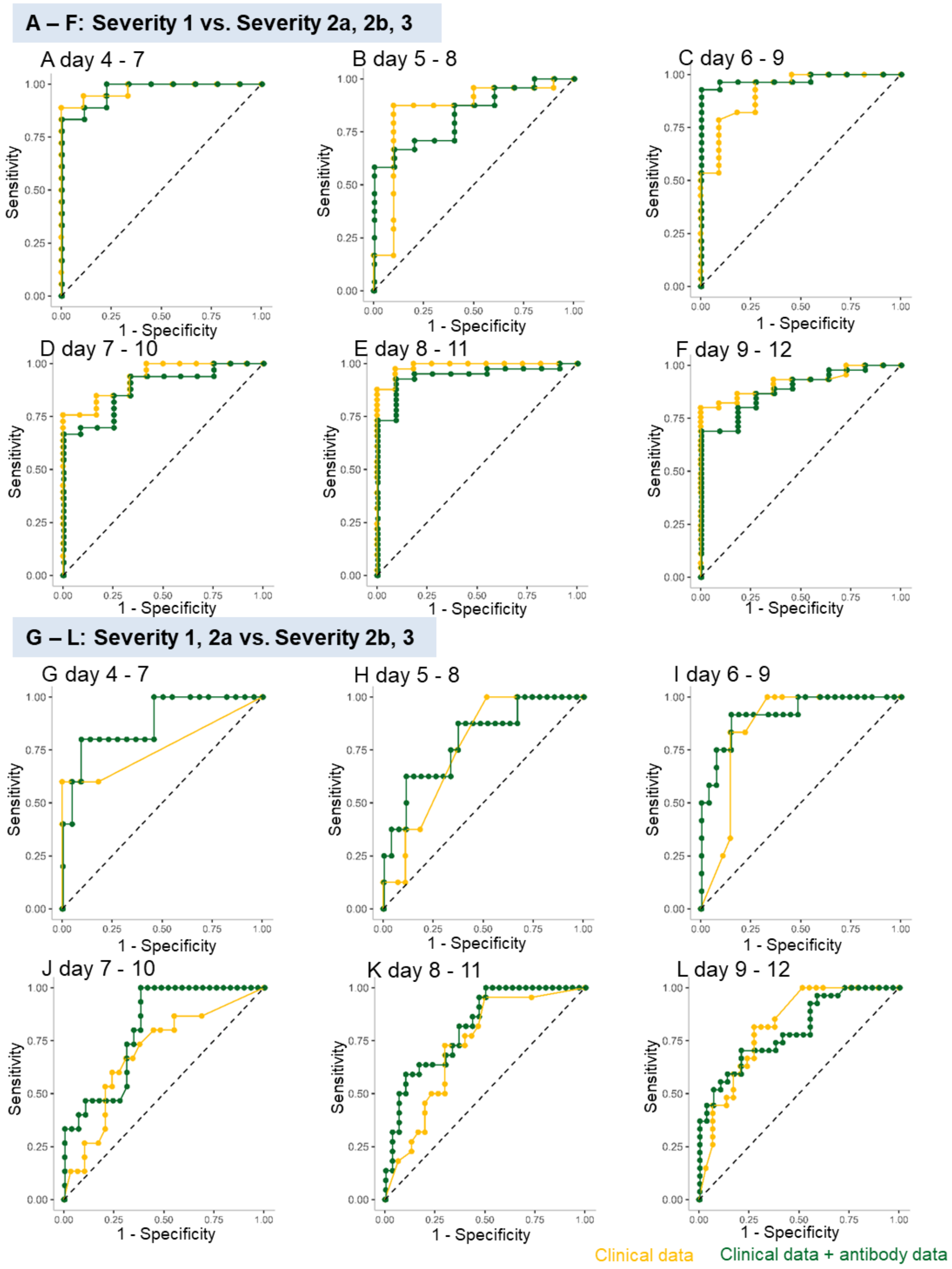

Supplemental Figure S10. ROC analyses of the analysis models constructed using a machine learning technique with only antibodies against N antigen for predicting the severity of COVID-19.
The ROCs of the analysis models constructed using a machine learning technique for predicting the COVID-19 severity, using the data obtained on day 4–7 (A, G), day 5–8 (B, H), day 6–9 (C, I), day 7–10 (D, J), day 8–11 (E, K), and day 9–12 (F, L), are shown. The models were constructed to distinguish severity groups 2a or over from severity group 1 (A–F) or distinguish severity groups 2b and 3 from severity groups 1 and 2a (G–L). The yellow curves represent the ROCs of the model constructed using clinical parameters and the green curves represent those of the model constructed using both clinical and antibody data (IgM(N), IgG(N), and IgA(N)).

Supplemental Figure S11

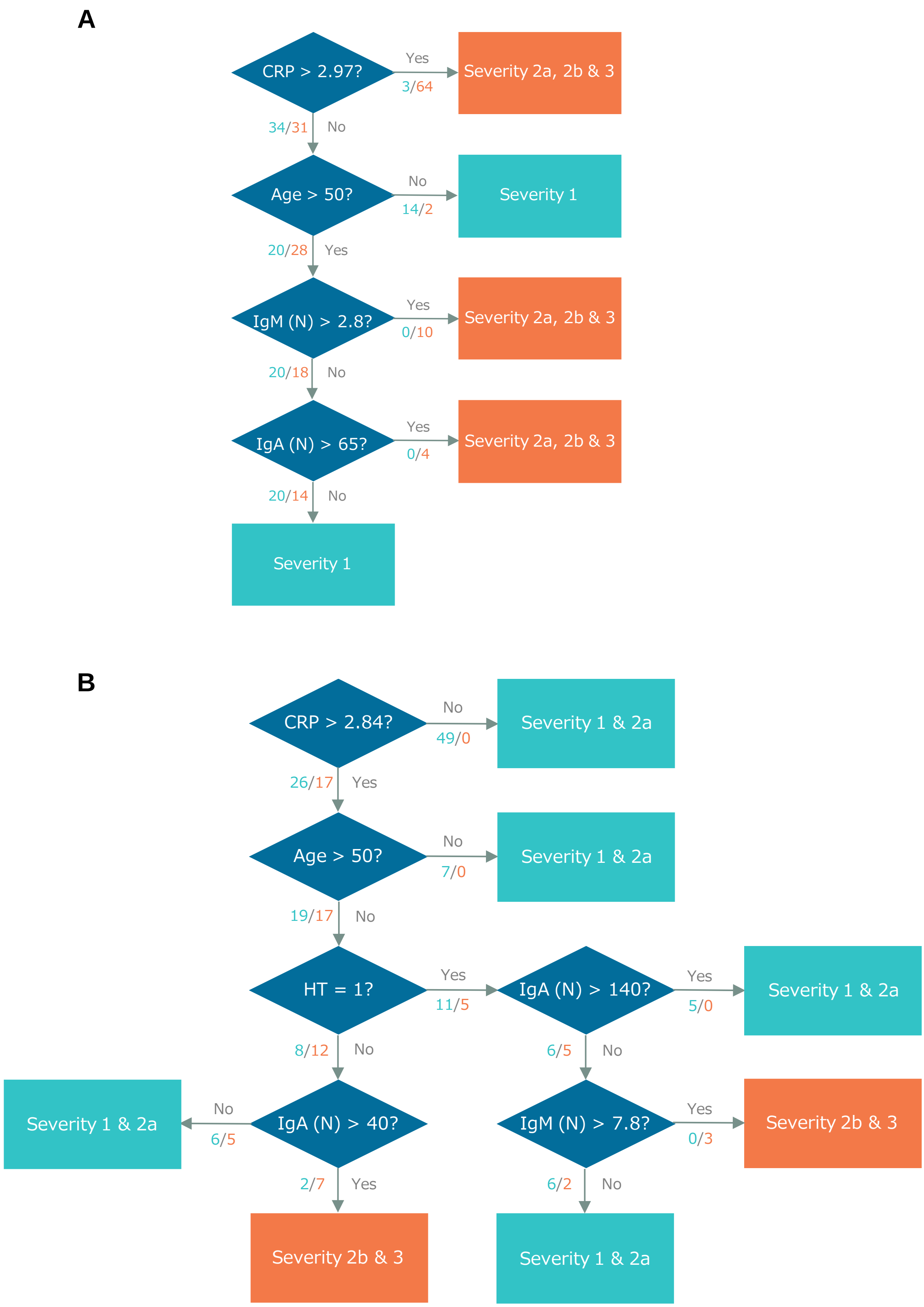

Supplemental Figure S11. The workflow to predict severity groups of 2a or over, which represents one of tree estimators in the optimum model, on day 6-9 (A) and that to predict severity group 2b or 3, which represents one of the tree estimators in the optimum model on days 4-7 (B).

Supplemental Figure S 12

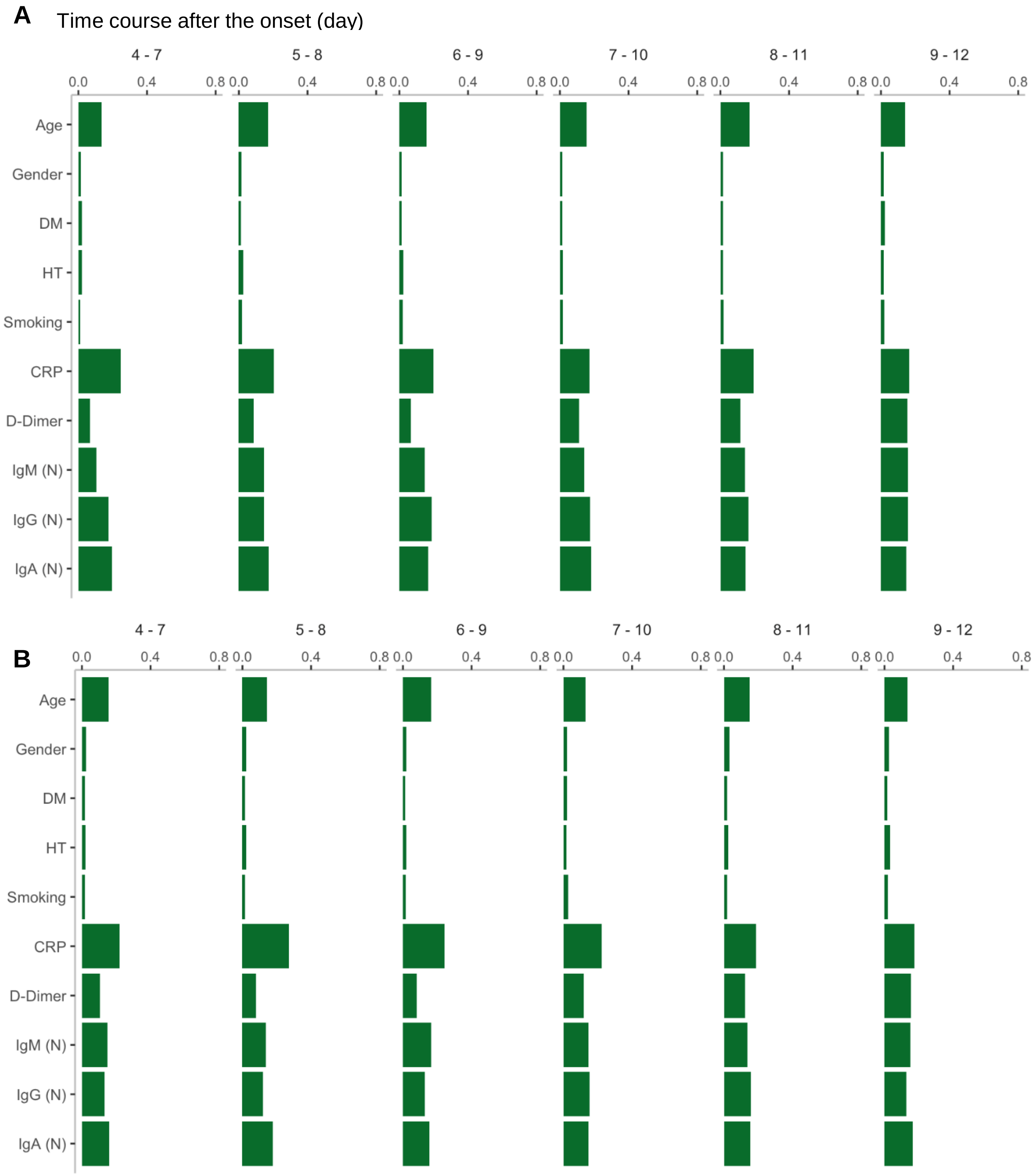

Supplemental Figure S12. The feature importance in the model constructed using a machine learning technique with the clinical data with the antibody data of antibodies against N antigen to distinguish severity groups of 2a or over and severity group 1 (A) and to distinguish severity groups 2b and 3 from severity groups 1 and 2a (B).
